# Functionalized poly(glycidylmethacrylate) for selective uranium(vi) adsorption: experimental and theoretical calculation insights

**DOI:** 10.1039/d5ra08591h

**Published:** 2026-02-02

**Authors:** Ahmad A. Tolba, Ebrahium Abdel Gwad, Marwa M. Rashad, Zeinab M. Shalaby, Walaa A. Kassab, Nilly A. Kawady, Said E. Mohammady, Ahmed H. Orabi

**Affiliations:** a Nuclear Materials Authority P.O. Box 530, El-Maadi Cairo Egypt A_orabi_chem@yahoo.com

## Abstract

This study investigates the recovery of uranium(vi) using a novel functionalized polyglycidyl methacrylate (PGMA) adsorbent, PPA-PGMA, modified with polyamine-phosphonic acid. The adsorbent's structure was confirmed by CHNP, BET, SEM, TGA, XRD, XPS, and FTIR analyses. Batch adsorption studies from synthetic solutions revealed an optimal pH range of 3.0–6.0, where the saturation adsorption capacity reached 0.828 mmol g^−1^. The adsorption process exhibited fast kinetics (180 min) and was endothermic. Experimental data fitted well with the Langmuir and pseudo-second-order (PSO) kinetic models. The adsorption process was quantitatively described using a new three-dimensional (3D) nonlinear mathematical model, which was verified using MATLAB software against several theoretical models (generalized Langmuir, PSO with Arrhenius, shrinking core, and Floatotherm models). Thermodynamic analysis indicated a spontaneous (Δ*G* < 0) and endothermic (Δ*H* > 0) reaction. The adsorbent demonstrated excellent reusability, maintaining high efficiency over six cycles. Metal desorption was successfully achieved using NaHCO_3_, with adsorption capacity remaining at 88–90% of the initial value after the sixth cycle. Finally, PPA-PGMA was applied to recover U(vi) from acidic ore leachates (El-Sella and Gattar areas) following precipitation pre-treatment. The adsorbent exhibited marked selectivity for U(vi) over co-existing Fe and Si, achieving adsorption capacities of 0.71 mmol U per g (El-Sella) and 0.65 mmol U per g (Gattar). These results confirm the potential of PPA-PGMA as a durable and selective adsorbent for uranium recovery from complex acidic matrices.

## Abstract 2

This study investigates the recovery of uranium(vi) using a novel functionalized polyglycidyl methacrylate (PGMA) adsorbent, PPA-PGMA, modified with polyamine-phosphonic acid.

## Graphical abstract

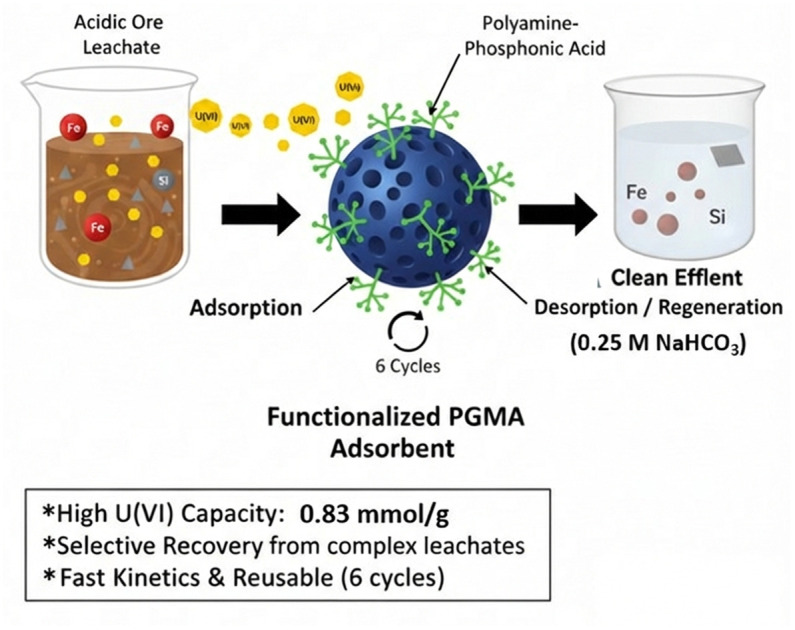

## 1. Introduction

A variety of energy sources are necessary due to the growing and continuous need for power, which makes energy a crucial component of sustainable development.^1–3^ Economic progress is increasingly dependent on nuclear energy, a typical efficient and sustainable energy source. When uranium, the main nuclear energy source, is discharged into the environment, it poses a health risk to the general population.^2,3^ Concerns about the environmental problems brought on by the handling and disposal of spent nuclear fuel are steadily growing.^1^ In radioactive wastewater, uranium usually exists in the form of uranyl ions (UO_2_^2+^). U(vi) possesses high solubility and mobility, making it easily absorbed and enriched by aquatic organisms and transmitted to higher organisms through the food chain, posing substantial risks to both ecological environment and human health.^3–5^ Thus, it is essential to separate and collect uranium from mine and industrial wastewater in order to reduce any risks to environmental security and to recover uranium.^1,2,4–6^ Up to today, numerous technologies have been utilized to deal with waste from industries.^6^ For the treatment of diluted metal solutions, traditional methods like solvent extraction or precipitation have technical or financial constraints, whereas mineral sorbents,^1,7^ chelating resins and ion-exchange^8,9^ could be more suitable. More significantly, because of its great effectiveness and ease of use on a wide scale, adsorption has been shown to be among the most successful techniques for extracting and recovering UO_2_^2+^ from wastewaters.^1,3,4,6^

Herein, the extremely reactive monomer glycidyl methacrylate (GMA), which has vinyl and epoxy functionalities, satisfies the criteria for post-polymerization and chemical-modification.^10–12^ Generally, the anchored and attached molecules have N-, O-, S- or P-atoms, or a mixture of these elements, which serve as the basic centers for complexing cations and enabling selective separation.^9,12,13^ By similarity with synthetic polymers/resins,^12,14^ organophosphorus derivatives are recognized as effective metal complexing agents^15^ and used as industrial chemicals in metal extraction,^8^ water treatment,^12^ or pollution control.^11,14^

It is possible to complex and chelate materials using N-, P-, and/or O-donor ligands, either individually or in combination. These ligands serve as coordinating groups and exhibit highly selective extraction capabilities due to their unique steric arrangement and coordination geometry. These sorbents have been the subject of several investigations as beneficial materials with certain functional groups that have a special affinity for uranyl ions.^16–18^ For the chelating characteristics, the Pearson's Hard and Soft Acid–Base Theory (HSAB) is carefully followed.^19^ Uranium is more reactive and has a greater affinity for hard bases, such amine groups, than it does for carboxylic groups since it is a strong acid.^16,20^ By interacting with chelating agents that have O, P, and N functions, the diverse range of reactive groups found on many sorbents may explain their general attraction to uranyl species.^16^ Based on the Hard–Soft Acid–Base (HSAB) concept, multidentate N-donor ligands—including 5-azacytosine (Acy),^20^ polyamine,^18,21,22^ amidoxime groups,^23,24^ aminophosphonate,^16,25^ and bis-aldehyde functionality^26^—have demonstrated noticeable selectivity toward U(vi) sorption. Recent advancements in computer technology have made it simpler to use the nonlinear regression optimization approach in place of figuring out the values of isothermal, kinetic, and thermodynamic parameters. Because simulation modeling provides lucid insights into complicated systems, it offers useful solutions as well as offers various advantages that improve productivity, accuracy, and innovation. Using virtual models to simulate real world situations allows us to predict how a reaction would function under various scenarios without the need for physical prototypes. This saves money by reducing the number of physical tests required. Whereas algorithms and equations are used in computer-based simulation modeling offers a dynamic environment for their study, including the ability to see them in 2D or 3D. Additionally, simulation makes it possible to iterate more quickly, which speeds up design modifications and optimizations. By spotting any problems early in the design process, it increases product reliability, which eventually results in higher quality, faster, and safer finished products. It also encourages creativity by allowing the testing of novel concepts without the danger or expense of actual experiments.^27,28^ The importance of kinetics utilization for the chemical reaction yield describes the procedure and rate of sorption of an adsorbate (UO_2_^2+^/U(vi)) onto a certain adsorbent. For components, the Vermeulen model provides an explicit multicomponent adsorption model; like binary or ternary system, that obeying the Langmuir isotherm equation in a mono system adsorption case. Consequently, the Langmuir isotherm can be expanded using Taylor expansion series and modified to produce the modified Vermeulen model.^27,28^

In this study, we aimed to synthesize and utilize a high-performance micron-sized functionalized polyglycidyl methacrylate (PGMA) adsorbent, PPA-PGMA, incorporating a polyamino-phosphonic ligand for selective uranyl ion (UO_2_^2+^) adsorption. A detailed characterization of the material's physicochemical features was performed. We then quantitatively analyzed the adsorption process through rigorous studies on nonlinear isotherm, kinetics, and thermodynamics, leveraging advanced MATLAB simulations to model the data. The work concludes with an assessment of PPA-PGMA's regeneration and recycling capacity and its practical application for uranium recovery from acidic ore leachates.

## 2. Materials and methods

### 2.1. Materials

Wako Chemical Co. Ltd (Japan) supplied the following: ethanol (99.5%), polyvinylpyrrolidone (PVP K-30), formaldehyde solution (37%), diethylenetriamine (DETA), glycidyl methacrylate (GMA), and 2,2-azobisisobutyronitrile (AIBN). We received phosphoric acid from Sigma-Aldrich (Darmstadt, Germany). A stock solution of 1000 mg per L uranium was used to create uranium(vi) ion solutions at various concentrations. Initial and equilibrium uranium concentrations were determined by spectrophotometry using a UV-visible spectrophotometer (Metertech Inc., model SP-8001) and the Arsenazo III colorimetric approach.^29^ Each extra chemical was bought from Prolabo Corporation (France) and used precisely as directed.

### 2.2. Adsorbent preparation

The detailed synthesis of the parent PGMA and the sequential functionalization with poly(phosphonic amine) were previously reported^8,10,11^ and were given in the SI.

### 2.3. Adsorbent characterization

The characterization of the adsorbent involved several analytical techniques. A Micro Corder JM10 analyzer (J-Science Lab Co., Ltd, Tokyo, Japan) was used for elemental analysis. FT-IR spectra were collected using an FT/IR-6600 spectrometer (JASCO, Tokyo, Japan). X-ray diffraction (XRD) patterns were obtained using a Smart Lab X-ray Diffractometer (RIGAKU, Tokyo, Japan) with Cu Kα radiation. Thermal deterioration behavior was observed through TG/DTA research utilizing an EXSTAR 6000 TG/DTA 6300N thermogravimetric temperature analyzer. The sorbent's textural properties were tested using a Quantachrome Nova 3200 surface area analyzer (USA) after the sorbent had been degassed for three hours at ambient temperature. The specific surface area (*S*_BET_) was calculated using N_2_ adsorption/desorption isotherms, and the BJH technique was used to assess the porous volume. X-ray photoelectron spectroscopy (XPS) was performed using a Japanese ESCA-5600 ESCA spectrometer. The point of zero charge (pH_PZC_) of the sorbent was determined by the pH-drift technique.^30^ Samples were equilibrated in a series of 0.1 M NaCl solutions of varying initial pH values and shaken for 24 h. The pH_PZC_ was identified as the pH at which the initial and equilibrium values were equal (pH_i_ = ΔpH_eq_).

### 2.4. Metal sorption and desorption

Batch adsorption experiments were performed by contacting 0.01 g of the functionalized chitosan-based sorbent with 20 mL of uranium(vi) solution (initial concentration, *C*_o_ = 50 mg U per L) in 30 mL glass bottles (AS One). The suspensions were agitated at 200 rpm and maintained at 25 ± 1 °C for 3 h. After separation, the residual uranium concentration (*C*_eq_, mg U per L) was determined, and the equilibrium adsorption capacity (*q*_eq_, mg g^−1^) was calculated using: *q*_eq_ = (*C*_o_ − *C*_eq_)*V*/*m*, where *m* (g) is the sorbent mass and *V* (L) is the solution volume. The distribution coefficient (*K*_d_) was determined as: *K*_d_ = [(*C*_o_ − *C*_eq_)/*C*_eq_]*V*/*m*.

Equilibrium was typically reached within 180 min under standard conditions (pH = 4, *T* = 25 ± 1 °C). Each experiment was repeated two or three times, and the relative standard deviation did not exceed 6%. The influence of pH, contact time, adsorption isotherms, temperature, desorption, and sorbent recyclability were systematically investigated, as detailed in the figure captions.

Sorbent regeneration and reusability were evaluated over six consecutive adsorption–desorption cycles. For each run, 20 mg of sorbent was treated with 40 mL of a 250 mg U per L solution for 180 min at room temperature. Following adsorption, the loaded sorbent was rinsed with deionized water and subjected to desorption using 5 mL of 0.25 M sodium bicarbonate solution for 1.5 h with continuous stirring. The uranium concentration in the eluate was analyzed, and desorption efficiency (*D*_E_) was calculated according to: *D*_E_ = *C*_D_ × *V*(L) × 100/*q*_d_ × *m*_d_, where *C*_D_ (mg L^−1^) is the uranium concentration in the desorption solution, *V* (L) is the desorption volume, *q*_d_ (mg g^−1^) is the adsorbed amount prior to desorption, and *m*_d_ (g) is the sorbent mass used. The adsorption capacity after each cycle was compared with the initial value to assess regeneration performance.

## 3. Results and discussion

### 3.1. Adsorbent synthesis and characterization

**Scheme 1:**
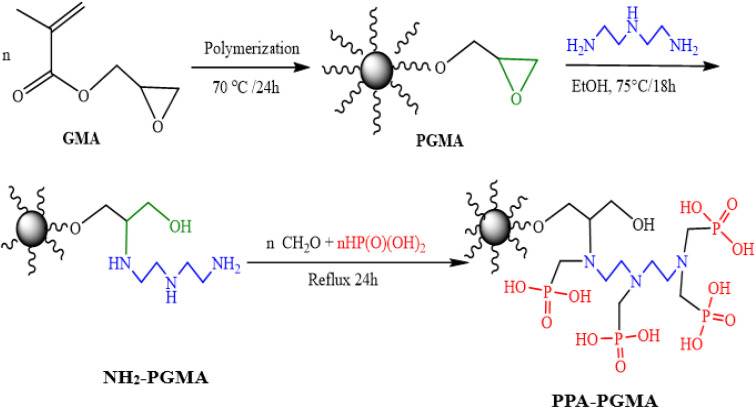
Synthesis of poly aminophosphonic acid-functionalized polyglycidyl methacrylate (PGMA): PPA-PGMA.

The synthesis of polyaminephosphonic acid-functionalized polyglycidyl methacrylate (PGMA), or PPA-PGMA, is schematically depicted in Scheme 1. Following the dispersion polymerization method's production of parent PGMA (micro-particles <75 µm), diethylenetriamine is used to open the epoxide ring, and amino groups are grafted.^10,31^ Finally, phosphonic acid groups react with amine functions *via* formaldehyde to graft methylenephosphonic groups onto the intermediate product. Both mono- and di-substituted amines may be present in the grafted polymer (for example, polymer –NH–CH_2_ –PO_3_H_2_ and/or polymer –N(–CH_2_–PO_3_H_2_)_2_) because the addition of a methylene phosphonic group to a chitosan macromolecule that contains phosphorous acid and formaldehyde may cause one or two phosphonomethyl moiety for polysubstituted structure to produce different derivatives.^8,11^ The identification of these reactive groups has been approached using a variety of analytical approaches.

### 3.2. Adsorbent characterization

#### 3.2.1. Morphology of PGMA particles and its textural features

Fig. S1a shows a SEM picture of PGMA particles. Massive items of varying sizes are depicted in this image. The distribution of particle sizes, which can be seen in the same graphic, may be evaluated using the image program (Foxit PhantomPDF). The particles have an irregularly shaped with partial aggregation. It is determined that the average particle size is 52.92 ± 6.61 µm. Fig. S1b displays the XRD patterns of the virgin and functionalized PGMA materials. Poorly resolved XRD signals are consistent with the PGMA substrate's weak crystallinity. The amorphous PGMA's XRD fingerprint shows a strong peak at 2*θ* = 17.7° and weak, broad shoulders at 2*θ* = 9.5–13°, 28–31°, and 40–44°.^32^ The relative decrease in the intensity and width of the XRD peaks confirms that the substitution of PGMA *via* tetraethylenepentamine and aminophosphonomethylation may increase the disorder in the polymer structure, even though the general structure is preserved (peaks are detected at angles close to the values reported for pristine PGMA). N2 adsorption/desorption isotherms have been used to investigate the textural properties of functionalized PGMA (PPA-PGMA) (Fig. S1c). According to Langmuir classification, the data can be classified as Type II isotherms. Notably, the profile displays a very weak hysteresis loop, which may be regarded as mesoporous (that is, the PPA-PGMA does not exhibit significant surface porosity). For single point and multipoint analysis, the specific surface area (*S*_BET_: in m^2^ g^−1^) is around 24.96 and 50.54 m^2^ g^−1^, respectively, with an average pore width of about 2.52 nm. Additionally, the 0.0438 cm^3^ STP per g pore volume. This magnitude is in line with the SSA values of PGMA that have been published. Wei *et al.*^33^ reported an *S*_BET_ for PGMA that was almost 29 m^2^ g^−1^, and the *S*_BET_ rose when the divinylbenzene co-polymer concentration increased.

#### 3.2.2. pH zero-point charge (ZPC)

The pH_PZC_ values of PGMA, NH_2_-PGMA, and PPA-PGMA were determined using the pH-drift method (Fig. S1d). The stepwise modification significantly influenced the acid–base properties of the polymers. Incorporation of amine groups through DETA grafting increased the pH_PZC_ from 5.9 to 8.43, consistent with the basic nature of DETA (p*K*_a_ = 3.58, 8.86, and 9.65).^34^ Similar pH_PZC_ values (∼8.2) have been reported for DETA-functionalized graphene oxide.^35^ Subsequent phosphonomethylation markedly reduced the pH_PZC_ to 2.65 due to the introduction of acidic phosphonate groups (p*K*_a_ ≈ 2.35). In comparable systems, such as chitosan functionalized with methyl or phenyl aminophosphonate derivatives,^25^ opposite trends in pH_PZC_ have been observed, where phenyl substitution lowered the pH_PZC_ to 3.13 while methyl substitution increased it to 6.24. The substantial drop in pH_PZC_ after phosphonomethylation in the present study suggests the replacement of the most basic amine groups, implying nearly complete substitution.

Aminophosphonic acids typically exhibit two dissociation constants: a strong acidic proton (p*K*_a_1__ ≈ 0.5–1.5) associated with the –P–OH group and a weaker acidic proton (p*K*_a_2__ ≈ 5–6) linked to the amine function.^36^ This amphoteric character results from intramolecular hydrogen transfer between P–OH and –NH_2_ groups. The electron-withdrawing nature of the P=O moiety reduces electron density on nitrogen, lowering its basicity.^36^

For ion-exchange resins containing aminomethylphosphonic groups, such as ES 467,^37^ protonation and deprotonation occur as follows: at low pH (∼1), the amine group is protonated, and one P–OH is deprotonated, forming a zwitterionic structure; at neutral pH (∼6), the phosphonate hydroxyls are largely deprotonated; and at high pH (∼11), both amine and phosphonate groups are fully deprotonated. These behaviors correspond well with the titration profile observed for PPA-PGMA seen in Fig. S1d.^25^ The acid–base characteristics of the functionalized polymer therefore govern its electrostatic interactions with metal ions, as well as competitive proton exchange and chelation processes during adsorption.

#### 3.2.3. Elemental analysis

The conversion of weight percentages for C, H, and N constituents in molar units, as per the theoretical structure of PGMA, suggests that the polymer can be addressed using the heptameric formula: (C_7_H_10_O_3_)_7_. Comparing the product's elemental analysis (CHN analysis) at different production phases allows one to track the chemical alteration of PGMA (Table S1).^10^ The nitrogen content rises to 12.46% (w/w or 8.90 mmol N per g) after the reaction with the polyamine (diethylenetriamine, DETA) due to its immobilization. Comparing the theoretical nitrogen proportions in DETA and NH_2_-PGMA allows one to determine the substitution degree close to 63%. The intermediate product (NH_2_-PGMA) is grafted with methylene phosphonic groups when phosphonic acid groups react with amine functions in the presence of formaldehyde. 1.582 mmol P per g, or 4.9% of the sorbent's weight percentage, is the P concentration. By chemically grafting on the matrix backbone, the materials' C and N mass fractions are rationally decreased; in the final PPA-PGMA, the N content decreases from 12.46% to 9.48% (w/w). The C and H mass fractions also fall at the same time; the C content drops significantly from 42.85% to 34.72% (w/w) to a level similar to that of PGMA material. Assuming the phosphomethylation process continues as illustrated in Scheme 1, a secondary amine with one phosphomethyl moiety or a primary amine with two phosphomethyl moiety can graft amine groups. The estimated result (*i.e.*, 9.77%) for a derivative grafted with only one phosphonate moiety on the polymer backbone H_2_PO_3_ : R–N ratio is comparable to the relevant weight fraction of N observed in the product, 9.48%. In reality, 1.67 P mole per mole of NH_2_-PGMA would be the simulated value. It is possible to mimic grafting 1–4 phosphomethyl groups on NH_2_-PGMA. The chemical investigation indicates that the N/P molar ratio in PPA-PGMA is around 4.28. This demonstrates unequivocally that the amine groups have not been completely substituted. Although phosphomethylation is effective, most amine groups remain free because the resin has two functions: it associates reactive amine and phosphomethyl groups.

#### 3.2.4. FTIR analysis

**Fig. 1:**
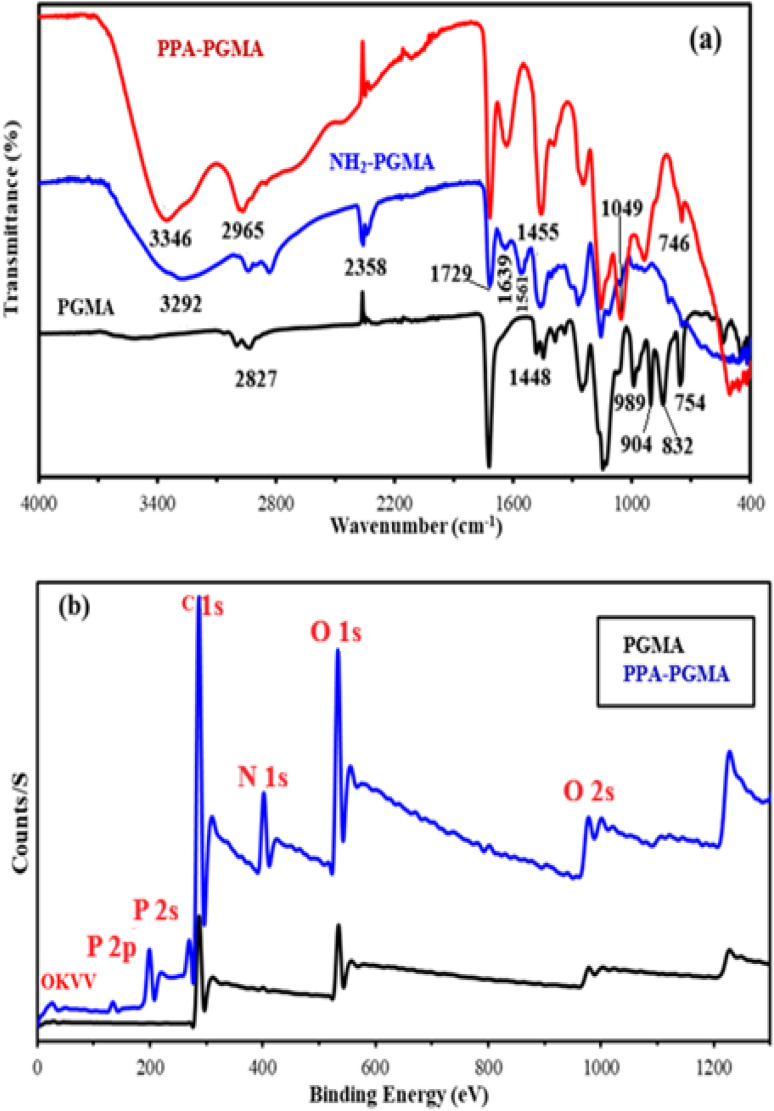
FTIR spectra of PGMA, NH_2_-PGMA and PPA-PGMA (a) and XPS survey for the raw PGMA, and PPA-PGMA (b).

The further chemical modification of PGMA (after the grafting of DETA and the final grafting of phosphonomethyl groups) can be monitored using FTIR spectroscopy. FTIR spectroscopy can also be used to identify the functional groups involved in metal binding. Characteristic band shifts, appearances, or disappearances may be linked to these alterations.^38,39^ The FTIR spectrum development for the various synthesized materials and metal-loaded PPA-PGMA is shown in Fig. 1a. The most significant groups that can be utilized to determine the fingerprint of the polymer are the carbonyl groups (*ν*(–COO–)) at 1729 cm^−1^,^40^ and the oxirane ring, which has asymmetrical stretching and expansion vibrations at 832 cm^−1^ and 904 cm^−1^, respectively.^12,41,42^ Another peak, about 754 cm^−1^, has also been ascribed to epoxy rings.^43^ The peaks have been attributed to C–O stretch vibrations in the 1300–1100 cm^−1^ range.^43^ Following DETA-grafting, the bands characteristic of the epoxy ring in the FTIR spectrum of NH_2_-PGMA vanish,^44^ but the peak at 1729 cm^−1^ is still discernible. This suggests the grafting is carried out by opening the epoxy ring and that the chemical alteration has little effect on the carbonyl groups. New bands at 1561 cm^−1^ and 1639 cm^−1^, on the other hand, are caused by N–H bending modes in secondary and primary amines, respectively.^44,45^ PGMA resin changed by grafting ethylene diamine^41^ and cellulose modified first with PGMA and then by extra grafting of polyethyleneimine (PEI),^42^ both showed similar changes. The presence of N–H groups causes further, poorly resolved alterations to be seen at around 3346 cm^−1^ (Fig. 1a). The peaks associated with the epoxy ring (at 904 cm^−1^, asymmetric vibration of epoxy ring) do not exist, which further validates how the amine compound was grafted onto PGMA.^44^ When NH_2_-PGMA reacts with phosphorous acid in the presence of formaldehyde, the final change results in the introduction of new P-based reactive groups. This is supported by typical bands at 746 cm^−1^, which are attributed to *ν*(–P=O), 932 cm^−1^, which are credited to P–O–C stretching^45^ or P–OH stretching,^46^ 1049 cm^−1^, which are connected to P–O–R bond, and 1244 cm^−1^, which are assigned to P=O bond.^47^ These new peaks validate the successful grafting of phosphorus-based reactive groups.

#### 3.2.5. XPS analysis

**Fig. 2:**
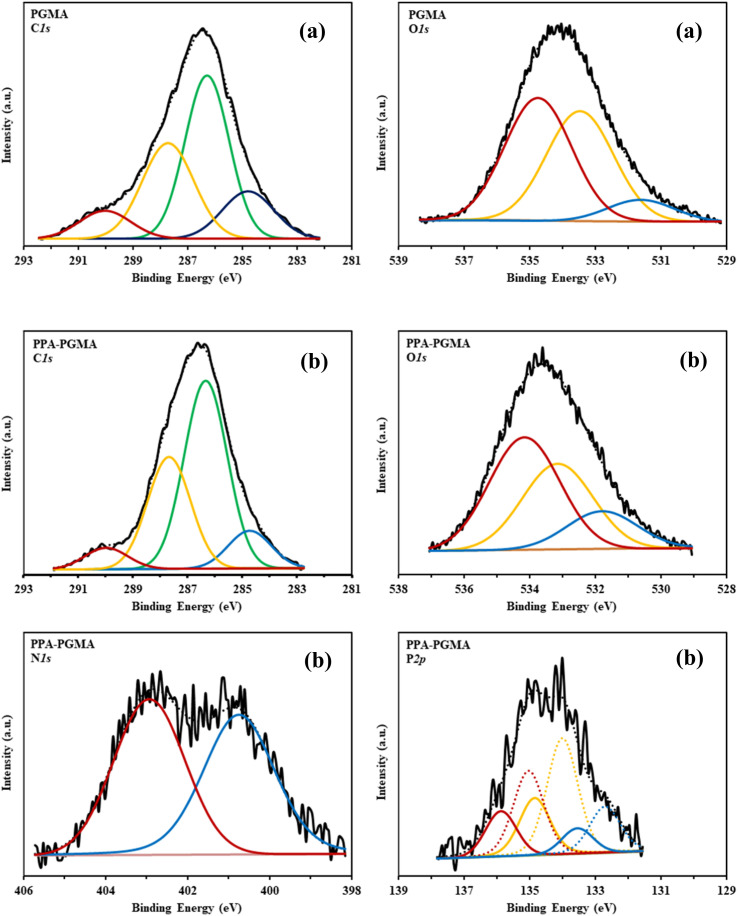
High resolution XPS spectra of C 1s and O 1s for PGMA (a) and C 1s, O 1s, N 1s and P 2p for PPA-PGMA (b) (solid black line: raw spectrum, black dotted line: composite spectrum, and colored lines: deconvoluted bands).

XPS measurements provided insight into the chemical modifications of PGMA during functionalization. Grafting of diethylenetriamine introduced nitrogen-containing groups, while subsequent phosphonomethylation incorporated phosphorus-bearing functionalities.^10^ High-resolution spectra of C 1s, O 1s, N 1s, and P 2p (Fig. 2 and Table 1) revealed characteristic binding energy shifts associated with these modifications.

**Table 1:** Analysis of XPS spectra (R: raw spectrum, BC: spectrum after baseline correction)

	PGMA		PPA-PGMA		Possible chemical assignments of BEs
	BE (eV)	AF (%)	BE (eV)	AF (%)	
C 1s	284.8	15.3	284.7	10.5	C–H, C–C, C_advent._
	286.3	44.9	286.3	52.3	C–O–C, C–NH, C–O–P
	287.7	30.8	287.7	31.1	C=O, C–P, C_epoxy_
	290.1	9	290	6.1	π–π* Sat., carbonate
O 1s	531.7	8.6	531.8	15.9	C=O, P–O, H_2_O
	533.5	43.1	533.2	36.3	C–O–C
	534.7	48.3	534.2	47.8	C–OH, P–OH
N 1s			400.8	49.6	C–N, –NH_2_, <svg xmlns="http://www.w3.org/2000/svg" version="1.0" width="10.400000pt" height="16.000000pt" viewBox="0 0 10.400000 16.000000" preserveAspectRatio="xMidYMid meet"><metadata> Created by potrace 1.16, written by Peter Selinger 2001-2019 </metadata><g transform="translate(1.000000,15.000000) scale(0.011667,-0.011667)" fill="currentColor" stroke="none"><path d="M80 1160 l0 -40 40 0 40 0 0 -40 0 -40 40 0 40 0 0 -40 0 -40 40 0 40 0 0 -40 0 -40 40 0 40 0 0 -40 0 -40 40 0 40 0 0 -40 0 -40 40 0 40 0 0 -40 0 -40 40 0 40 0 0 80 0 80 -40 0 -40 0 0 40 0 40 -40 0 -40 0 0 40 0 40 -40 0 -40 0 0 40 0 40 -40 0 -40 0 0 40 0 40 -40 0 -40 0 0 40 0 40 -80 0 -80 0 0 -40z M560 520 l0 -40 -40 0 -40 0 0 -40 0 -40 -40 0 -40 0 0 -40 0 -40 -40 0 -40 0 0 -40 0 -40 -40 0 -40 0 0 -40 0 -40 -40 0 -40 0 0 -40 0 -40 -40 0 -40 0 0 -40 0 -40 80 0 80 0 0 40 0 40 40 0 40 0 0 40 0 40 40 0 40 0 0 40 0 40 40 0 40 0 0 40 0 40 40 0 40 0 0 40 0 40 40 0 40 0 0 80 0 80 -40 0 -40 0 0 -40z"/></g></svg> NH
			402.9	50.4	NH_3_^+^, NH_2_^+^
P 2p			132.7	12.5	P–O (2p_3/2_)
			133.5	6.3	P–O (2p_1/2_)
			134	31.2	P–C, PO_4_, deproton phosphonate (2p_3/2_)
			134.9	15.6	P–C, PO_4_, deproton phosphonate (2p_1/2_)
			135	23	Proton phosphonate (2p_3/2_)
			135.9	11.5	Proton phosphonate (2p_1/2_)

Minor variations in the C 1s and O 1s regions confirmed limited substitution on oxygen-containing sites. The P 2p spectra displayed weakly resolved peaks corresponding to protonated and deprotonated phosphonate species. Peaks at 402.9 eV and 400.8 eV in the N 1s region indicated the coexistence of protonated and unprotonated amine species.

These results collectively demonstrate the successful incorporation of amine and phosphonate groups into the PGMA matrix and their active involvement in uranium coordination.

#### 3.2.6. Thermal properties

**Fig. 3:**
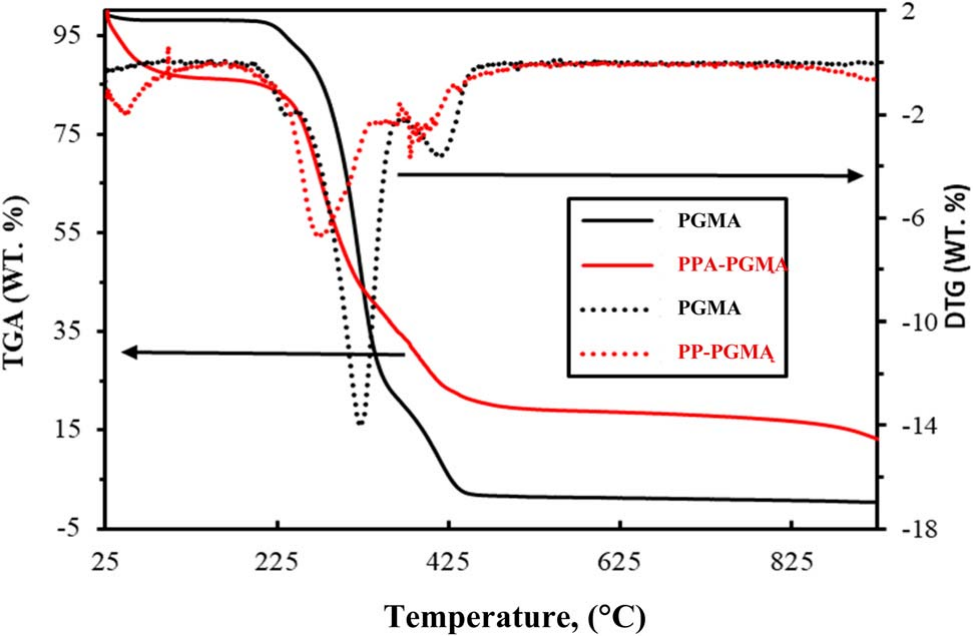
Thermal analysis: TGA (solid line) and DTG (dot line).

Significant differences exist between the degradation patterns of DTG and TGA (thermogravimetric analysis and differential thermogravimetry) (Fig. 3): PGMA's chemical transformation has been confirmed. PGMA's TGA profile shows many stages of degradation:

(a) A stable plateau below 210 °C, when the release of absorbed water is accompanied by a weight loss of no more than 2–3%.

(b) There was a noticeable decrease in weight between 210 and 344 degrees Celsius (with the highest rate of loss seen on the DTG curve at 320°); this is somewhat in line with the traditional TGA thermogram of PGMA,^48^ which showed that the largest rate of degradation was at 301 degrees Celsius. Random chain scission is blamed for the degeneration. About 70% of the weight has been lost.

(c) PGMA loses 27% of its total weight between 344 and 440 degrees Celsius (highest loss rate on DTG at 410°); this is due to the breakdown of the polymer's shorter chains, which results in the development of a limited char (less than 3%).

(d) In order to achieve total deterioration, PGMA gradually loses the residual char portion over 440 °C and up to 825 °C.

Because PPA-PGMA contains novel organic chains with both amine and phosphonic groups, the thermal breakdown profile is more complicated:

(i) The more hydrophilic modified polymer releases absorbed water between 25 and 65 degrees Celsius (less than 14 percent weight loss).

(ii) A steady plateau between 65 °C and 230 °C with a small weight loss (about 3%), most likely brought on by the release of constitutive water (or more securely linked to polymer chains).

(iii) The breakage of amine chains or the degradation of the sorbent by polymer scission may be the cause of the notable weight reduction about 38%, with a maximum loss rate at 274 °C on DTG seen in the 230 °C to 320 °C range.

(iv) A fresh weight loss of 21% of the total mass is noted between 320 and 430 degrees Celsius. This is attributed to the creation of char and the breakdown of phosphonate moieties.

(v) While 13% (w/w) of the solid is still present at 925 °C, the char progressively breaks down at 430 °C and up to 925 °C. For flame-retardant properties, phosphorus-based compounds are frequently used as char-forming additives because they promote char formation and partially block polymer scission.^49^

The chemical alteration promotes char formation, improves water absorption, and improves heat stability (more residual solid and lower slope of weight loss). Furthermore, the degradation profiles demonstrate that the thermal deterioration starts at a lower temperature (shift of around 30 °C) than with raw PGMA.

#### 3.2.7. Interaction mechanism

##### 3.2.7.1 FTIR analysis

After U(vi) adsorption (Fig. S2a), the intensities of the bands at 3357 and 2966 cm^−1^ decrease noticeably, indicating alterations in the local chemical environment of hydroxyl and amine groups due to metal binding and partial overlap of poorly resolved vibrational modes. Overall, the FTIR spectrum of the U(vi)-loaded resin remains similar to that of the pristine material, with several distinct changes observed upon metal uptake. Specifically, the band at 503 cm^−1^ exhibits a blue shift to 531 cm^−1^, which is attributed to the formation of U–N coordination bonds. In addition, a new absorption band appears at 633 cm^−1^, corresponding to metal–oxygen (M–O) bond formation, while another band at 826 cm^−1^ is assigned to M–CO interactions. Meanwhile, the band originally observed at 932 cm^−1^ disappears after uranium loading. A prominent new band emerges at 1310 cm^−1^, which falls within the characteristic region of C–N stretching vibrations in amine groups,^45^ confirming the involvement of nitrogen donor atoms in metal coordination.

Furthermore, the chemical environments of nitrogen- and phosphorus-containing functional groups are significantly affected by U(vi) binding. The P=O stretching vibration, initially observed at 1049 cm^−1^, undergoes a red shift to 1038 cm^−1^ accompanied by a marked increase in intensity, indicating the participation of phosphonate groups in metal coordination. In the low-frequency region (400–800 cm^−1^), additional bands associated with M–OH vibrations and O–M–O stretching modes are detected, which are characteristic of metal–oxygen bonding environments commonly observed in layered double hydroxide-intercalated biopolymer nanocomposites.^45,46,50^

##### 3.2.7.2 XPS analysis

PPA-PGMAA was synthesized *via* polymerization of glycidyl methacrylate, followed by epoxy ring opening with a polyamine and subsequent phosphonomethylation using phosphorous acid and formaldehyde. The resulting polymer contains abundant phosphonic acid, amine, and hydroxyl functional groups, which provide multiple coordination sites for U(vi) adsorption.

XPS was employed to investigate the surface elemental composition and chemical changes of PPA-PGMAA before and after uranium adsorption. Fig. S2b shows the wide-scan XPS spectra of pristine and uranium-loaded PPA-PGMAA. The pristine material exhibits characteristic peaks of C 1s, O 1s, N 1s, and P 2p/P 2s, confirming the successful functionalization of the polymer matrix.

After uranium adsorption, distinct uranium-related signals appear, most prominently in the U 4d region (736–739 eV),^50^ which are completely absent in the pristine sample. The presence of the U 4d peak provides direct and unambiguous evidence for the successful immobilization of uranium species on the PPA-PGMAA surface. In polymer-based adsorbents, the U 4d core level is particularly suitable for uranium identification in survey scans due to its high photoionization cross-section and minimal overlap with other elemental signals. A weak feature overlapping with the N 1s region is observed; however, the U 4f doublet cannot be reliably resolved from the survey spectrum owing to signal overlap and the relatively low surface concentration of uranium. Nevertheless, the binding energy positions and strong intensity of the U 4d peaks are consistent with reported values for hexavalent uranium, indicating that uranium is predominantly retained as U(vi).

Following uranium adsorption, an increase in the O 1s signal intensity and minor changes in the relative intensities of the P 2p and N 1s peaks are observed, suggesting the involvement of oxygen- and nitrogen-containing functional groups in uranium coordination. These changes reflect alterations in the local chemical environment of phosphonic acid, hydroxyl, and amine groups upon metal binding.

Hydrochloric acid was used for pH adjustment during adsorption experiments; therefore, trace chloride species may coexist on the adsorbent surface. The Cl 2p was shown at approximately 198.3 eV and 199.6 eV, attributed to protonated amine–chloride ion pairs and weakly bound residual chloride species, respectively.^50^ After uranium adsorption, the Cl signal nearly disappears, indicating the release of chloride ions during metal uptake.

Additionally, metal sorption induces a shift of the O 1s binding energy from approximately 531.8 to 530.8 eV, accompanied by changes in relative peak intensities, further confirming the participation of oxygen-containing functional groups in uranium coordination.

##### 3.2.7.3 SEM-EDS analysis

The SEM micrograph of PP-PGMA after uranium uptake (Fig. S2c) reveals pronounced surface roughening and the development of denser domains compared to the unloaded adsorbent, indicating strong surface–metal interactions and partial coverage of active sites by uranium species. The corresponding EDS spectrum collected from the selected region confirms the presence of distinct uranium characteristic peaks, which are absent in the pristine PP-PGMA, providing direct elemental evidence for successful U(vi) immobilization on the adsorbent surface.

The XPS results are consistent with FTIR observations and support a mechanism in which phosphonic acid groups act as primary binding sites, assisted by neighboring amine and hydroxyl functionalities through multidentate surface complexation.

**Scheme 2:**
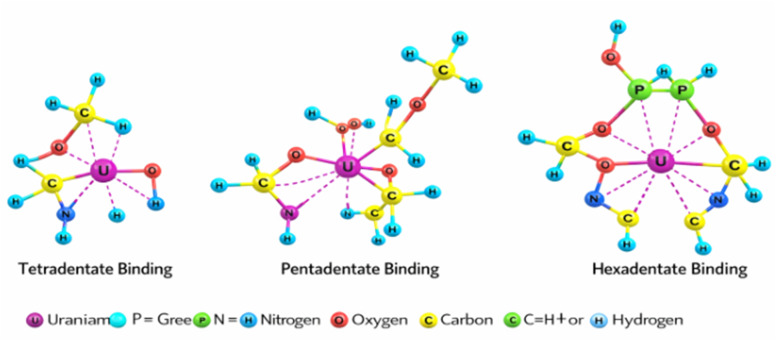
Proposed coordination structures of U(vi) with aminophosphonate ligand in PPA-PGMA.

Given the polymeric and heterogeneous nature of PPA-PGMA, U(vi) may adopt different equatorial coordination modes. Uranyl typically maintains a linear O=U=O axial geometry and coordinates 4–6 donor atoms in the equatorial plane. In this work, three plausible binding scenarios are proposed: (i) tetradentate (involving two phosphonate O donors, one amine N, and one hydroxyl O), (ii) pentadentate (addition of a second nitrogen donor), and (iii) hexadentate (full participation of phosphonate, amine, and hydroxyl groups). These modes differ based on local protonation state, steric accessibility, and ligand deprotonation equilibria, and they are all chemically reasonable for aminophosphonate ligands. The Scheme 2 illustrates these options. The stability of these structures is justified based on FTIR and XPS data, which confirm the involvement of both nitrogen and oxygen donor atoms from phosphonic, amine, and hydroxyl groups.

Regarding the apparent preference for N-coordination, a strong blue shift of the band at 503 to 531 cm^−1^ and the emergence of new CN bands at 1310 cm^−1^ suggest U–N complexation, while red-shifting of the P=O stretching band from 1049 to 1038 cm^−1^ indicates that P=O also participates in metal binding through deprotonated phosphonate oxygen atoms. Therefore, the coordination environment is mixed (N/O/P) rather than exclusively nitrogen-based. The revised text clarifies that P=O coordination contributes through a hard oxygen donor interaction, while tertiary amines act as neutral soft bases that stabilize uranyl through inner-sphere complexation. This is consistent with reported interactions of UO_2_^2+^ with aminophosphonate ligands and polyaminocarboxylate chelators.

### 3.3. Adsorption studies

Uranyl (UO_2_^2+^) adsorption was investigated using the synthesized adsorbent's properties, starting with the impact of pH and uptake kinetics. To evaluate thermodynamic characteristics, adsorption isotherms were computed at different temperatures. Lastly, metal recycling and desorption experiments were performed.

#### 3.3.1. Effect of pH

**Fig. 4:**
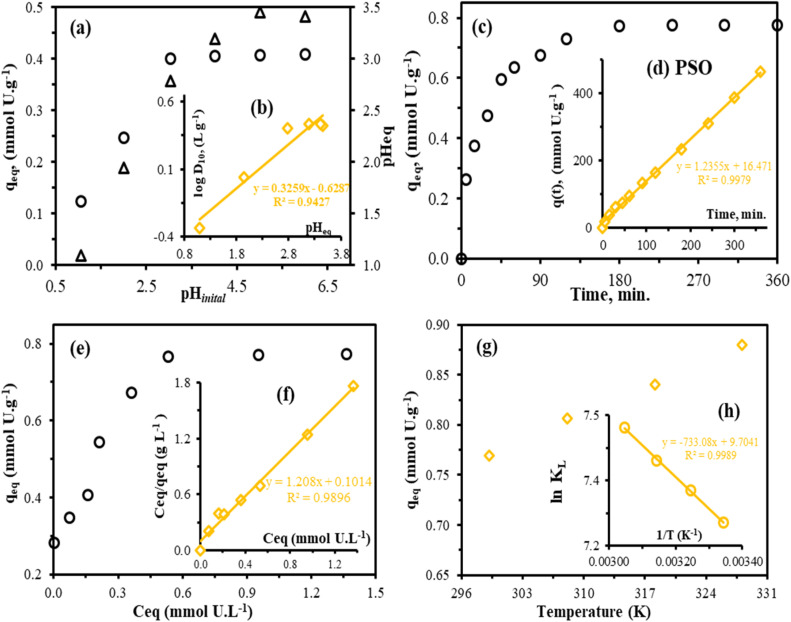
Effect of pH adsorption: sorption capacity *vs.* pH _initial_ (a), plot of log *D*_10_*vs.* pH_eq_ ((b), inserted), uptake kinetics (c), PSO plot ((d), inserted), (d) adsorption isotherms (e), Langmuir isotherm ((f), inserted), temperature effect (g), and thermodynamics of adsorption–Van't Hoff plots of ln *k*_L_*vs.* 1/*T* ((h), inserted).

A common factor that influences metal species in solution, metal ion sorption on sorbents, and the surface properties of sorbents in terms of surface charges and dissociation of functional groups is the pH of a solution.^2,10^ A PPA-PGMA batch adsorption experiment using U(vi) ions ([M] ∼ 50 mg L^−1^) at different pH values (1.0–6.0) revealed that the pH significantly impacted the adsorption of metal ions. Because of its positively charged surface, which repels metal cations by an attraction force, PPA-PGMA exhibited minimal adsorption when the pH was below 2. The reduction in adsorption capacity is caused by competition between U(vi) and H(i) ions at the various active centers of the adsorbent. The H(i) ion has a high adsorption competition due to its small size.^51^ PPA-PGMA reached *q*_eq_: 0.41 in mmol U per g and shown the best adsorption performance for U(vi) in the pH range of 3.0–5.5 (Fig. 4a). The exchange or interaction of free metal ions with PPA-PGMA active sites is probably the cause of this.^52^ Reactive groups (hydroxyl, phosphonate, and amino groups in different materials) prefer to deprotonate when pH_0_ rises over ZPC, which is compatible with the ZPC-values and the metal–ligand interaction.^28^ This change decreases electrostatic repulsion or promotes attraction with the positively charged U(vi) species, allowing for enhanced metal–ligand coordination and a resulting increase in adsorption capacity.^53,54^ For U(vi) future research, the pH was therefore adjusted at 4.0.

The pH_0_ fluctuation following metal ion adsorption is seen in Fig. 4a. The pH_eq_ did not vary in the pH_0_: 1.0–2.0 range, however it did fluctuate and trend to drop at pH_0_: 3.0–6.0 (where the pH stabilizes at ∼3.3). This “buffering effect” aligns with PPA-PGMA's acid-base properties.^28^ The distribution ratio's log plot (*D*: *q*_eq_/*C*_eq_, L g^−1^) against pH_eq_ is shown in Fig. 4b. In an ion-exchange process, the slopes of the log *D*_10_ plot *vs.* pH_eq_ connection are linked to the stoichiometric anion/cation exchange with bound metal species. As seen in Scheme 2, the slope is around 0.3259 with an *R*^2^ of 0.9427, indicating that the ion-exchange mechanism is significantly influencing the regulation of the adsorption process.^28^

#### 3.3.2. Effect of contact time and kinetic studies

**Fig. 5:**
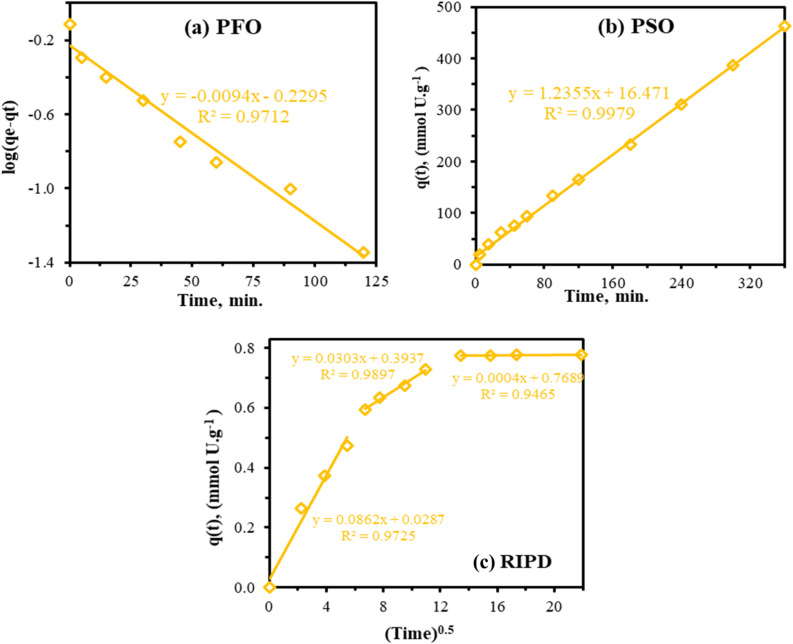
Kinetic models (a) PFO, (b) PSO, and (c) RIPD models for U(vi) ions adsorption.

The adsorption kinetics was analyzed using PPA-PGMA adsorbent with U(vi) at concentration ([U]_0_ ∼ 0.84 mmol L^−1^). The PPA-PGMA adsorption kinetics is extremely fast with ∼49% in the first 15 min and ∼93.2–99.8% of the capacity was attained in 180 min (Fig. 4c). The subsequent 180 minutes showed a slower uptake, followed by adsorption equilibrium, reaching *q*_m_ = 0.774 mmol U per g, demonstrating the effectiveness of PPA-PGMA as an adsorbent. The adsorption data as a function of time was evaluated using the intra-particle diffusion models, pseudo-first-order kinetic (PFO), and pseudo-second-order kinetic (PSO) models (Fig. 5) to gain a better understanding of the adsorption kinetics (the mathematical equations are shown in Table S2, see SI).^55^ The experimental adsorption data may also be fitted to these kinetic models to determine their parameters. Table 2 summarizes the kinetic parameters that were determined by fitting these models. The experimental data suited the PSO kinetic model best (Fig. 4d), as demonstrated by comparing the adsorption capacities (*q*_m_, calculated and experimental, Table 2): the PSO model differences overestimated value (4.60%), whereas the PFO data underestimated values (23.75%). Furthermore, the correlation coefficient (*R*^2^) is as follows: PSO > *R*^2^ (0.997) > PFO. Considering that the experimental data best suit the PSO, chemical adsorption is projected to be the rate-determining phase.^54,56^ The resulting *k*_2_ values were used to compute the half-sorption time (*t*_0.5_), or the time required for the ion-exchange resins to reach 50% of their maximal sorption capacity. As shown by the label (*t*_0.5_ = 1/*k*_2_*q*_2_),^53,57,58^ the *t*_0.5_ could be utilized as a measure of the adsorption rate and was 13.3 min.

**Table 2:** Kinetics and isotherm constants adsorption

Kinetics			Isotherms		
**PFO**			**Langmuir isotherm**		
*K*_1_ × 10^−3^ (min^−1^)	*q*_e,1_ a	*R* ^2^	*q*_m,L_ a	*K*_L_, L mmol^−1^	*R* ^2^
21.65	0.59	0.9712	0.828	11.9132	0.9896

**PSO**			**Freundlich isotherm**		
*K*_2_ × 10^−3^^b^	*q*_e,2_ a	*R* ^2^	*n*	*k*_F_ a	*R* ^2^
92.68	0.809	0.9979	6.68	0.7013	0.7739

**sRIDE**					
*K*_id,1_ c	*K*_id,2_ c	*K*_id,3_ c			
0.0862	0.0303	0.0004			

a Units: mmol metal per g.

b Units: g mmol^−1^ min^−1^.

c Units: mmol g^−1^ min^−0.5^.

According to Hubbe *et al.*,^59^ the kinetic profile compatibility provided by the PSO indicates that intraparticle diffusion governs the process. For rate control operations in the sorption process, the RIDM was evaluated using the Weber and Morris plots (Table 2 and Fig. 5), which are multi-linear plots with three primary phases.^60^ The first two main phases are the progressive adsorption stage and the external surface adsorption stage. This suggests that the adsorption rate is impacted by the boundary layer as well as intraparticle diffusion. The last adsorption phase is getting close to adsorbent saturation.^53,57,58^

#### 3.3.3. Adsorption isotherms

Solutions ([U]_0_ ∼ 0.11 to 1.67 mmol L^−1^) were examined at pH_0_ 4.0 and 298 K as a function of starting metal concentrations in order to assess adsorbent capabilities. The adsorption equilibrium results are shown in Fig. 4e. The equilibrium adsorption capacity (*q*_e_) significantly increased with rising initial and equilibrium metal concentrations. The capacity appeared to stabilize when the equilibrium concentration exceeded approximately 0.53 mmol L^−1^. This stabilization is likely attributed to the saturation of available adsorption sites on the PPA-PGMA sorbent surface.^54,56^ The maximum experimental adsorption capacity (*q*_m,exp_) achieved was approximately 0.74 mmol U per g.

**Fig. 6:**
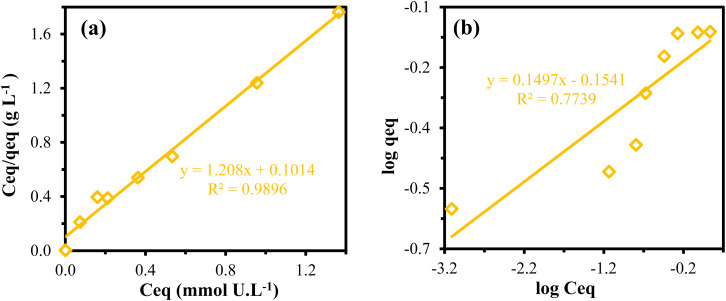
Isotherm models (a) Langmuir and (b) Freundlich for U(vi) ions adsorption.

The PPA-PGMA adsorption mechanism for metal ions was understood by fitting the adsorption data using the Langmuir and Freundlich models.^55^ An empirical theory known as the Langmuir isotherm makes the assumption that monolayer adsorption takes place on a uniform surface. In contrast, the Freundlich model is frequently used to represent the adsorption process that occurs on a heterogeneous surface.^54^ By fitting the experimental adsorption data with these models, the values of these isotherms' model parameters can be obtained (Fig. 6). The constants obtained by fitting these models are displayed in Table 2. The correlation coefficient values (*R*^2^) showed that the Langmuir model was more appropriate for the isotherm data than the Freundlich model. Additionally, the projected adsorption capacities were in excellent accord with the actual measurement, showing that the Freundlich model's values were underrated (Δ*q*_m,cal._ = 7.36–18.28%) while the Langmuir isotherm values were exaggerated (Δ*q*_m,cal._ = 3.71–7.32%). The findings imply that uranyl ion monolayer production occurred on the PPA-PGMA surface.

#### 3.3.4. Adsorption thermodynamics

To investigate the thermodynamic characteristics of different metal ion adsorption on PPA-PGMA, the adsorption capabilities were examined at four different temperatures, ranging from 299.15 to 328.15 K, at pH_0_: 4.0, and *C*_0_: 200 mg L^−1^ (Fig. 4g). The Van't Hoff equation was used to calculate the enthalpy change (Δ*H*°, kJ mol^−1^) and entropy change (Δ*S*°, J mol^−1^ K^−1^) as follows: ln *D* = (−Δ*H*°/*R*)1/*T* + Δ*S*°/*R*, while the free energy (Δ*G*°, kJ mol^−1^) change was computed from Δ*G*° = Δ*H*° − *T*Δ*S*°.^25,61,62^ Following dimensionless processing (which accounts for molality in the water) and non-corrected conversion (from L g^−1^ to L mol^−1^), Lima *et al.*^63^ calculate the distribution coefficients (*D*: *q*_eq_/*C*_eq_ in L g^−1^) at each temperature. The thermodynamic parameters are described in Table 3, and the linear plots of ln *D vs.* 1/*T* demonstrate a satisfactory fit with the experimental results (Fig. 4h). Regardless of the kind of cation, the absolute Δ*H*° values (which range from 5.68 to 6.09 kJ mol^−1^) exhibit positive signs, support the reaction's endothermic nature, and make the process more advantageous at higher temperatures. The increase in randomness following metal contact at the solid–liquid border is shown by the positive value of Δ*S*° (between 67.73 and 80.68 J mol^−1^); this is probably related to the water release (like the results from the water hydration of mercury species^64^). The sorption process appears to be spontaneous based on the negative values of Δ*G*°. The temperature is proportional to the absolute Δ*G*° values (which fall between −18.04 and −20.38 kJ mol^−1^).^2,65^Table 3 indicates that entropic changes, not enthalpy changes, have an impact on the reaction (|Δ*H*°| < |*T*Δ*S*°|).^28^

**Table 3:** Thermodynamic parameters of U(vi) ions adsorption

Temp., K	Δ*H*° (kJ mol^−1^)	Δ*S*° (J mol^−1^)	Δ*G*° (kJ mol^−1^)	*T*Δ*S*° (kJ mol^−1^)	*R* ^2^
299.15	6.09	80.68	−18.04	24.14	0.9930
308.15			−18.77	24.86	
318.15			−19.57	25.67	
328.15			−20.38	26.48	

#### 3.3.5. Adsorption with preference

**Fig. 7:**
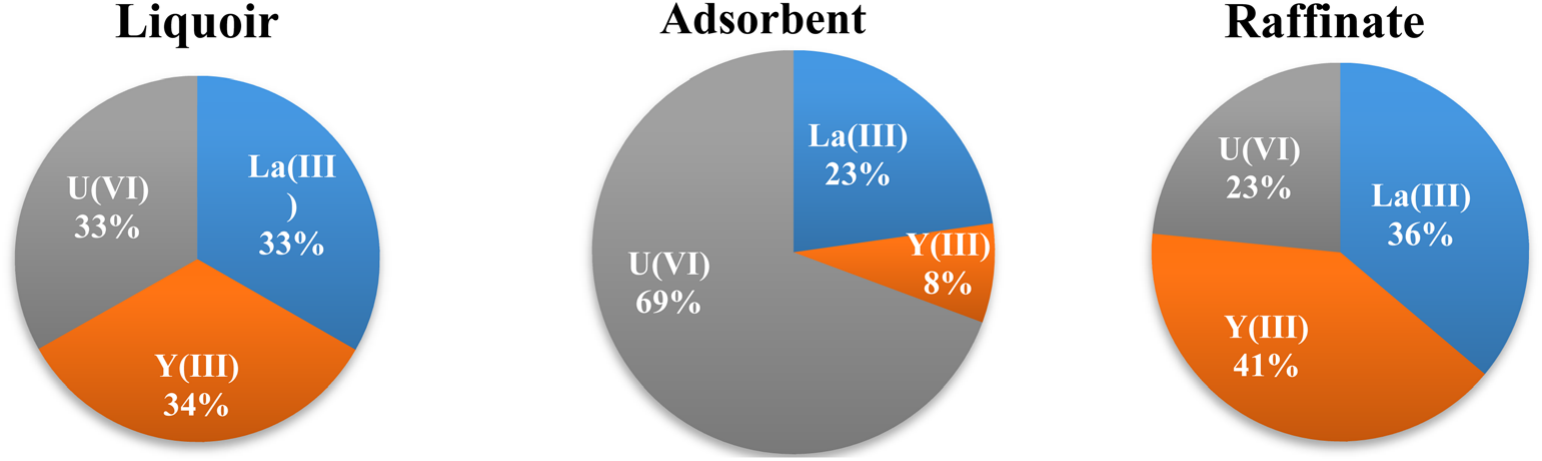
The distribution of the selected metal ions in liquor, treated raffinate, and the PPA-PGMA (molar percentages).

Since it appears difficult to separate individual elements, especially for REEs, both equilibrium and dynamic studies show that the PPA-PGMA adsorbent has relatively comparable adsorption capabilities for the three elements (U(vi)) and RE(iii) from distinct light (La(iii)) and heavy (Y(iii)) REEs families. A complementary experiment was conducted on a tertiary solution that included uranium (U(vi): ∼118.82 mg L^−1^), light REE (La(iii): ∼69.53 mg L^−1^) and heavy REE (Y(iii): ∼44.78 mg L^−1^) at equimolar quantities (*i.e.* ∼0.5 mmol L^−1^). The adsorption capabilities reach U(vi) (0.556 mmol U per g) > La(iii) (0.182 mmol La per g) > Y(iii) (0.064 mmol Y per g) at metal-binding equilibrium. The initial molar ratio of U(vi)/La(iii)/Y(iii) in the solution was about 1.0; following metal adsorption, the sorbent's molar ratio of La(iii)/Y(iii) rose to 1.21. The corresponding molar percentages of several co-metal ions in treated raffinate and the metal ions adsorbed onto the PPA-PGMA adsorbent surface are shown in Fig. 7. The solution before and after adsorption differed significantly. This is the first indication that uranium has a very strong selectivity, and that La(iii) and Y(iii) showed a little difference and concentration impact in the adsorbent. The percentages in the adsorbent show a variety of tendencies as compared to the leachate's initial composition: La(iii) mmol g^−1^ represented about 74.19% of the overall REs capacity (*i.e.*, 0.246 mmol g^−1^), which represented approximately 30.7% of the whole capacity (*i.e.*, 0.801 mmol g^−1^). U(vi) also showed a considerable enrichment and concentration (from 33.0% to 69.0%).

Even though RE(iv) was enriched in the solution, the changes were not particularly noticeable, and more enrichment stages would have been needed to achieve metal separation. The overall adsorption capacity was around 0.801 mmol metal per g, which is comparable to the highest adsorption capacities discovered for U(vi) binding (refer to Table 2). The metal ions fought for the same reactive groups, as was to be expected.

#### 3.3.6. Metal-desorption and recycling

Costs associated with metal recovery can be significantly decreased by the ability to efficiently reuse adsorbent material over a large number of adsorption/desorption cycles.^54^ To test PPA-PGMA's reusability in many re-use cycles, it was first equilibrated with U(vi) solution and then desorbed off using 0.25 M of NaHCO_3_. According to Table 4, the PPA-PGMA is reusable with a little reduction in metal adsorption capability following six reuse cycles, demonstrating its significant durability and straightforward regeneration process. The adsorption and desorption efficiencies are also higher than 91%. The stable sorption/desorption efficiencies observed post-six cycles indicate good chemical stability, as further confirmed by FTIR analysis (Fig. S3). Notably, the FTIR spectra of the PPA-PGMA sorbent without U(vi), closely resemble that of the recycled material after six cycles.

**Table 4:** PPA-PGMA adsorption desorption efficiency

Cycle no.	I^a^	II	III	IV	V	VI
Adsorption, %	100	97.5	96.02	94.15	92.57	91.08
Desorption, %	98.08	96.57	95.02	93.32	92.44	91.53

a Reference value for metal ion sorption efficiency (at first cycle).

### 3.4. Nonlinear adsorption studies in 3D

The equilibrium uptake of U(vi) can be affected by numerous variables; one of the most important one is the pH factor that has a direct impact upon the adsorption process and initial concentration of metal ions as well as contact time is another considerable factor in the adsorption process that impacts the process's economic efficiency and the adsorption kinetics.^64^ On the other hand, the solution temperature has a huge influence to change the nature of adsorbents causing expansion of its particles followed by mobility alteration of adsorbate ions and the solid/liquid interface, which controlled the rate of reaction within its vital role to describe the type of an exhibited interaction. To understand the U(vi) adsorption behavior, the chemical reaction must be reached to the equilibrium state for each factor affecting the reaction.^5^

#### 3.4.1. The relation between equilibration time–temperature

**Fig. 8:**
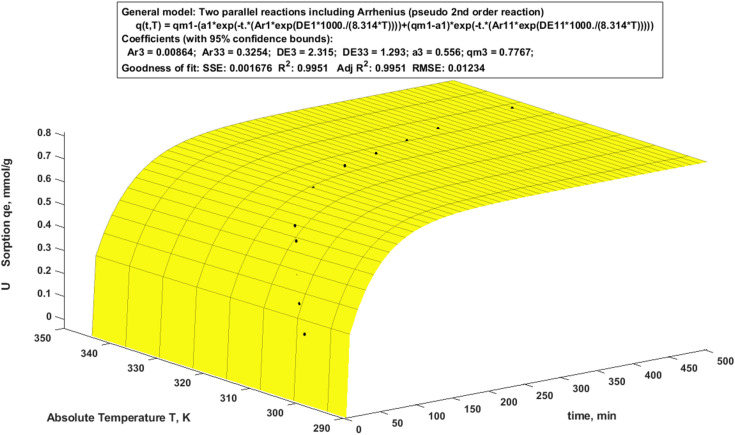
Two parallel reactions including Arrhenius (pseudo 2nd order reaction).

Modeling of experimental kinetic data provides a description of the uptake mechanisms and phenomena that control the adsorption process. Fig. 8 displays the adsorption capacity–time–temperature 3D experimental findings graphed using MATLAB. The applied nonlinear fitted equation of the PSO including Arrhenius was expressed as a function of time dependent and temperature (eqn (1)).^66^

1 *q*(*t*, *T*) = *q*_m1_ − (*a*_1_ × exp(−*t* × (Ar_1_ × exp(DE_1_ × 1000/(8.314 × *T*)))) + (*q*_m1_*a*_1_) × exp(*t* × (Ar_2_ × exp(DE_2_ × 1000/(8.314 × *T*)))))

The suggested model (eqn (1)) predicted that there are two chemical reactions were occurred following the PSO model including Arrhenius (Fig. 8) with a satisfactory correlation coefficient.^28^ Table S3 displays all parameters in Fig. 8. The easier adsorption kinetics is, the lower the *E*_a_ (activation energy). Notably, Ar_2_ > Ar_1_ confirms that the chelation mechanism is a quicker process than ion-exchange one.^28^

The level in which the mathematical findings coincide with the experimental results is measured by the error percentage (error % = ((*q*_e,exp._ − *q*_e,calc.1_) × 100)/*q*_e,exp._), which is used to assess the validity of the mathematical technique. The results of the mathematical equation's validation and comparison are shown in Table S3. The accuracy and reliability of the mathematical approach is evaluated and found that it is at maximum ±1.4343–0.0144%. The calculated uptake correction of U was evaluated, by applying the general model that combined Gauss and Fourier using the MATLAB software, that achieved more fitting with lesser error % (eqn (2)) (Table S3).

2 *q*_*t*_Ucor__ = *a*_1_ × exp(−((*x* − *b*_11_)/*c*_11_)^2^) + *a*_1_ × cos(*x* × *w*) + *b*_1_ × sin(*x* × *w*) + *a*_02_ + *a*_2_ × cos(*x* × *w*) + *b*_2_ × sin(*x* × *w*) + *a*_3_ × exp(−((*x* − *b*_3_)/*c*_3_)^2^)

#### 3.4.2. Determination of reaction type

Modeling of experimental data for the heterogeneous reactions is commonly used since Levenspiel 1999 postulation of a new model known as a shrinking core model.^27^ The primary purpose of using the shrinking core model is to identify the sort of reaction that happened based on its mechanism. As shown in eqn (3)–(5), three distinct regulating mechanisms occurred: (i) diffusion control through a product layer, (ii) surface response control, and (iii) diffusion control through a liquid film layer.^67^3
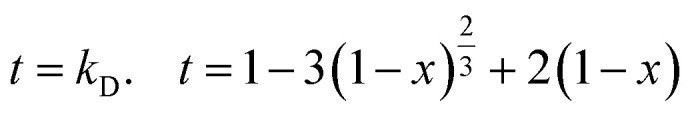
4
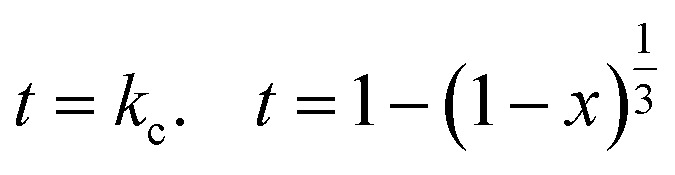

5 *t* = *k*_f_. *t* = *x*

where *X* is the proportion of particles that reacted, *t* is the reacted time, and *k*_D_, *k*_c_, and *k*_f_ are the reaction rate constants.

**Fig. 9:**
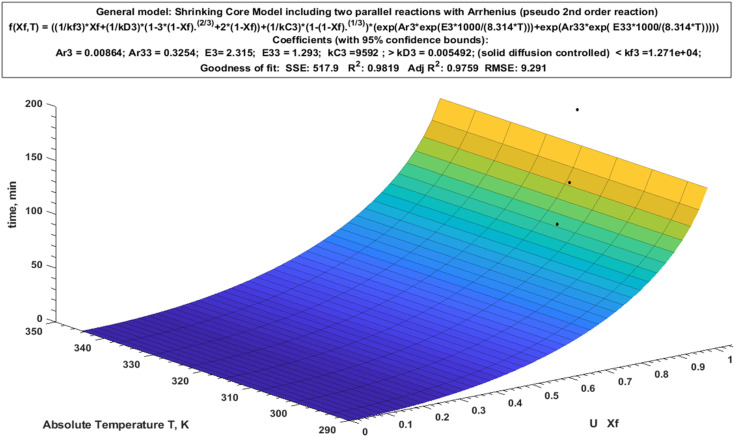
Shrinking core model including two parallel reactions with Arrhenius (pseudo 2nd order reaction).

The value of *k* used in the subsequent computation, which is derived from the best model and yielded the greatest determination coefficient (*R*^2^), was used to identify the reaction mechanism. The predicted nonlinear model was fitted from the 3D relation of *X*_f_ = (*q*_*t*_/*q*_m_) against time and absolute temperature (Fig. 9) as in eqn (6). According to Table S4, the reaction constant values for U follow the pattern *k*_c_ (9592)> *k*_D_ (0.00055) < *k*_f_ (1.271 × 10^4^), with *R*^2^ value (0.9819), indicating that the reaction is being regulated by a solid diffusion reaction.^27^ On the other hand, the rate constants for the chemical reaction (*k*_c_), solid diffusion reaction (*k*_D_), and film diffusion reaction (*k*_f_) are as follows. In this case, the solid diffusion response cripples the rate of reaction; thus, it is necessary to reduce the size of the adsorbent, increase its surface area and/or porosity, and speed up agitation in order to counteract that effect.

6 *f*(*X*_f_, *T*) = ((1/*k*_f_) × *X*_f_ + (1/*k*_D_) × (1 − 3 × (1 − *X*_f_)^{(2/3)}^ + 2 × (1 − *X*_f_)) + (1/*k*_c_) × (1 − (1 − *X*_f_)^{(1/3)}^) × (exp(Ar_1_ × exp(*E*_1_ × 1000/(8.314 × *T*))) + exp(Ar_2_ × exp(*E*_2_ × 1000/(8.314 × *T*)))))

#### 3.4.3. Thermodynamic analysis

**Fig. 10:**
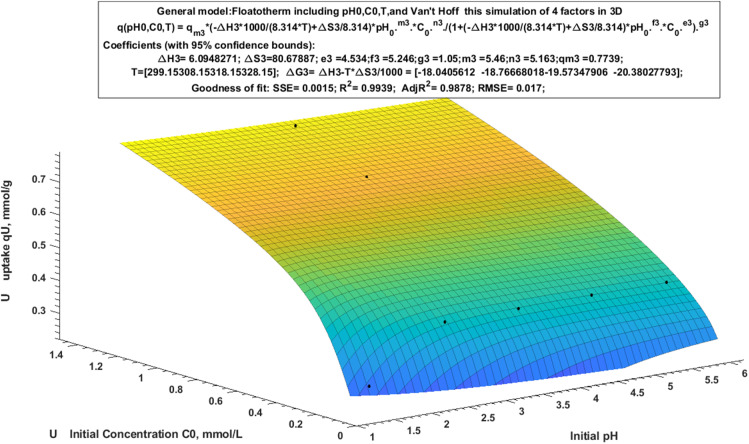
General Van't Hoff-Floatotherm model (anisotherm).

Other thermodynamic features are revealed when analyzing an adsorption process in three dimensions using the modified Langmuir equation (Fig. 10). The Van't-Hoff connection was added to the generalized Langmuir form to construct the Floatotherm model (eqn (7)). The thermodynamic parameters were then computed and shown in three dimensions using it.

7 *q*(pH_0_, *C*_0_, *T*) = *q*_m_ × (exp(−DH × 1000/(8.314 × *T*) + DS/8.314)) × pH^m^_0_ × *C*^n^_0_/(1 + (exp(−DH × 1000/(8.314 × *T*) + DS/8.314)) × pH^f^_0_ × *C*^e^_0_)^g^

Table 7 demonstrated the calculated adsorption capacity and the most important parameters of thermodynamics, with different initial pH (pH_0_) and different initial concentration (*C*_0_) (mmol g^−1^) at different temperature (K). The positive sign of Δ*H* refers to an endothermic reaction and its magnitude was about 6.0949 kJ mol^−1^. Likewise, the negative sign of Δ*G* values (in kJ mol^−1^) is related to a spontaneous reaction. On the other hand, Δ*S* (80.6788) kJ mol^−1^ K^−1^ is an important parameter to demonstrate by the fact that raise in entropy taken place because metal ion exchange increases ion mobility during the adsorption process. All these data are in agree with the experimental data.

Moreover, to create an automatic control system must be evaluating the correlation factor, which has the uptake values as close as possible to the reality. In this work, by drawing the relation between the measured uptake experimentally against the simulated uptake by utilizing the Gauss model to obtain the lowest possible value of error percentage. The correlation of U uptake was calculated as shown in Table S5 by applying general model Fourier (eqn (8)):

8 *f*(*x*) = *a*_0_ + *a*_1_ × cos(*x* × *w*) + *b*_1_ × sin(*x* × *w*) + *a*_2_ × cos(2 × *x* × *w*) + *b*_2_ × sin(2 × *x* × *w*).

where *q*_exp_ is an experimental uptake, *q*_calc1_ is the calculated uptake with respect to two variables (pH_0_ and *C*_0_), *q*_calc2_ is the calculated uptake in relation to three variables (pH_0_, *C*_0_, and *T*), and *x* = *q*_calc_, and *q*_cor_ is the corrected uptake.

### 3.5. Comparison with other adsorbents

Comparing the experimental results with those found in literature. The results of using PPA-PGMA adsorbent for uranium adsorption show that the adsorbent perform well as an effective adsorbent for removing uranium in comparison to the studies found in the literature (Table 5).

**Table 5:** Comparing the experimental results with those found in the literature

Adsorbent	pH	*q*_max_ (mmol U per g)	References
Amberlite CG-400	3.5	0.472	68
Phosphorus PStyr/DVB	5.0	0.378	69
DETA-magnetic chitosan	3.5	0.274	22
Carminic acid impregnated resin	5.0	0.798	70
D2EHPA-impregnated polymer beads	4.0	0.079	71
Amberlite IRA-402	3.0	0.895	72
Porous hydroxy apatite	3.0	0.468	73
Carboxylated-Zn-MOF	4.0	0.544	74
Amidoxime funct. catechol iron oxide NPs	6.5	0.256	75
IDA-PGMA	4.0	0.516	31
Algal/polyethyleneimine beads	4.0	0.150	50
PPA-PGMA	4.0	0.828	This study

### 3.6. Real sample application

#### 3.6.1. Application studies to El-Sella ore material sample

In Egypt's Southern Eastern Desert, the El-Sella mining region is situated around latitudes 22°14′30″–22°18′36″ N and longitudes 36°11′45″–36°16′30″ E. Abu Ramad (Red Sea Governorate) lies around 60 kilometers southwest of this place. The primary minerals found in this region include biotite, muscovite, plagioclase, and potash feldspar. It also identifies other secondary minerals such chlorite, kaolinite, and sericite. Ca(UO_2_)_2_(SiO_3_OH)_2_·5H_2_O and uranophane make up the majority of the secondary mineral uranium found in the G. El-Sella mining deposit. Since adding an oxidizing agent is not necessary to increase metal recovery, the fact that this uranium is already present in its oxidized state is especially important for acidic leaching.

To liberate uranium selectively, acidic agitation leaching was used. After the ore was ground, the proportion that corresponded to a particle size less than 100 mesh (<149 µm) was collected. The ratio of solids to liquids was set at 1 : 3, or 100 g of ore to 300 mL of sulfuric acid and ore leaching was carried out for 6 hours at 50 °C with agitation using a 50 g per L H_2_SO_4_ solution. After filtering the ore slurry, demineralized water was used to thoroughly sterilize the residue. To make the pregnant leach solution (PLS), the leachate and residual washes were mixed and adjusted to 300 mL with demineralized water. The PLS was then analyzed for base metal and U content. The leaching efficiency was 76.24% at a concentration of 298.21 mg U per L, according to the leaching experiment's data. The pH of the acid leachate was about 2.03. PLS was pre-treated with pH control using NaOH up to pH 3.5–4 (precipitated PLS, PPLS) to reduce the iron concentration and optimize the pH for U(vi) recovery.

In a batch reactor, PPLS was treated under experimental circumstances similar to those for synthetic solutions (pH_0_: 4; SD: 0.5 g L^−1^; duration: 3 hours; room temperature (*i.e.*, 55 ± 1 °C); and agitation speed: 200 rpm). The chemical analyses of the primary components of the ore processing are reported in Tables S6 and 6. These include the pregnant leaching solution (PLS), partially precipitation (PPLS, or precipitated pregnant leaching solution), adsorption, and pH regulation.

**Table 6:** Composition of the ore, acidic leachate (pregnant leaching solution, PLS), precipitated pregnant leaching solution (PPLS, pH control), treated solution after adsorption

Element	Reference oxide	Ore wt%	Metal concentration (g L^−1^)			*Q*_eq_		*D*, (L g^−1^)	SC, metal/U
			PLS	PPLS	PPA-PGMA	mg g^−1^	mmol L^−1^		
Si	SiO_2_	69.93	43.52	42.89	42.89	8.80	0.31	0.21	0.22
Al	Al_2_O_3_	13.13	18.09	17.90	17.89	12.40	0.46	0.69	0.76
Fe	Fe_2_O_3_	5.22	15.86	0.86	0.84	39.40	0.70	46.83	0.05
Ca	CaO	1.81	1.32	1.17	1.17	8.00	0.20	6.84	0.01
Mg	MgO	0.65	0.66	0.56	0.56	6.00	0.25	10.77	0.01
K	K_2_O	1.28	4.19	4.12	4.12	5.20	0.13	1.26	0.00
Na	Na_2_O	0.34	1.13	1.10	1.10	2.00	0.09	1.82	0.00
P	P_2_O_5_	0.69	1.03	1.02	1.02	4.60	0.15	4.53	0.00
Mn	MnO	0.23	0.33	0.31	0.31	6.80	0.12	21.96	0.02
Ti	TiO_2_	1.09	6.74	5.69	5.69	5.80	0.12	1.02	0.00
U	mg L^−1^	1173.4	298.21	269.09	184.46	169.26	0.71	917.60	1.00
LOI		5.41							
Total		99.78			Sum	268.26	3.25		

**Fig. 11:**
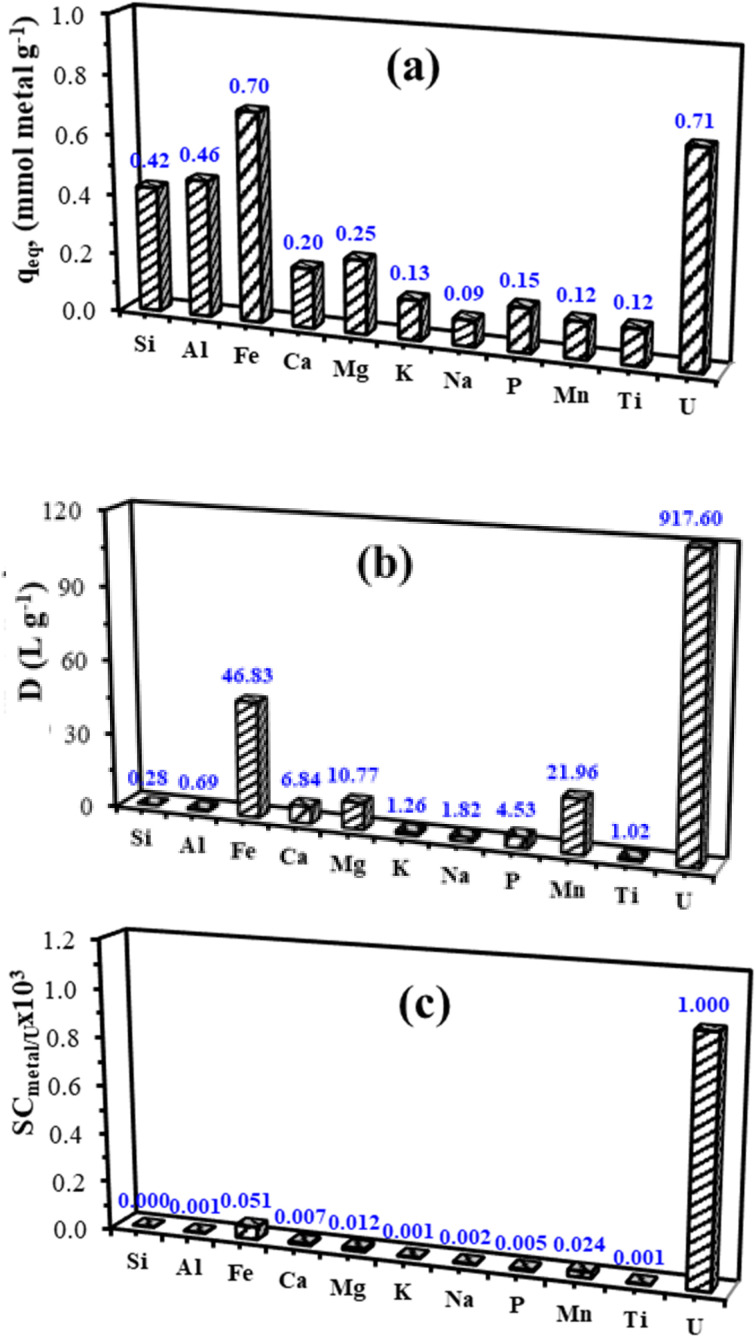
Selectivity tests: (a) adsorption capacities (in mmol metal per g), (b) distribution coefficients (*k*_d_ in mL g^−1^), and (c) selectivity coefficient (SC_metal/U_ × 10^3^) (pH_0_: 4.01; *T*: 55 ± 1 °C; SD: 0.5 g L^−1^; time: 3 h).

A surplus of metal ions in comparison to the quantity of sorbent utilized is the result of the experimental parameters used for the sorption experiments. When assessing the effectiveness of a therapy, the elimination efficiency is not regarded as a significant metric. Utilizing the data is done using the distribution of the ratio (*D*, mL mg^−1^), adsorption capacities (*q*_eq_, mmol g^−1^), and selectivity coefficient (SC_metal/U_ = *D*_metal_/*D*_U_) (Fig. 11). The adsorption capacity for U(vi), which is 184.5 mg U per g (*i.e.*, 0.71 mmol U per g), is much lower than the degree of saturation capacity evaluated on synthetic solutions, which is 197.06 mg U per g, or 0.828 mmol U per g (Fig. 11a).

This indicates that the maximum adsorption is reduced by ∼14% when high quantities of co-ions are present; when the significant excess of co-existing ions is taken into consideration, the sorbent exhibits a notable affinity for U(vi). Uranyl is in competition with some of the metal ions in the solution to bind to the particular reactive groups of the sorbent.

At 3.35 mmol metal per g, the overall adsorption capacity is higher than the maximum adsorption capacity for U(vi) in synthetic solutions. This is due to various stoichiometric ratios and/or different functional groups (such as non-substituted amine groups) that other metal ions attach to the particular reactive groups. PPA-PGMA is largely linked to just three metals: Si(iv) (0.42 mmol Si per g), Al (0.47 mmol Al per g), and Fe (0.71 mmol Fe per g).

Since the effective concentrations of the metals in PPLS clearly have a significant impact on the adsorption capabilities, the distribution ratio *D* is determined to more effectively highlight the sorbent's affinity for target metals (Fig. 11b). Therefore, compared to other co-ions, which have *D* values consistently lower than 49.2 mL g^−1^, Fe and U(vi) have much higher *D* values (46.8 and 917.4 mL g^−1^). PPA-PGMA has a tendency to favorably enrich U(vi) and Fe. The examination of selectivity coefficients (SC_metal/U_, Fig. 11c) confirms the effective preference of PPA-PGMA for U(vi). U(vi) is preferentially extracted from complicated acidic solutions by the adsorbent.

#### 3.6.2. Application studies to Gabal Gattar ore material sample

The northern eastern desert of Egypt is home to the Gabal Gattar mining area (longitudes 33°10′\–33°30′\E and latitudes 27°02′\–27°07′\N). The existence of many uranium deposits makes the Gabal Gattar granites noteworthy.^30^ The composition (major elements) of the ore is shown in Table S7; the uranium concentration of the ore can reach 801.43 g per ton. The composite head sample was subjected to chemical tests. The results of these studies, which are shown in Table S7, show that the head sample is mostly made up of feldspars and quartz, which are silicic gangue minerals that make up 88.0% of the oxides (SiO_2_ + Al_2_O_3_). The use of an acidic leaching solution is supported by these inert oxides. The following criteria were used to carry out acidic leaching: particle size: −200 mesh (<149 µm), solid/liquid ratio: 1 : 4, acid concentration: 20 g H_2_SO_4_ per L, U content: 801.43 g per ton, temperature: 50 °C, and mixing time: 6 hours. The leaching efficiency is approximately 94.4% at a concentration of 190.03 mg U per L (Table 7).

**Table 7:** Composition of the ore, acidic leachate (pregnant leaching solution, PLS), precipitated pregnant leaching solution (PPLS, pH control), treated solution after adsorption

Element	Reference oxide	Ore wt%	Metal concentration, mg L^−1^			*Q*_eq_, (mg L^−1^)	*Q*_eq_, (mmol L^−1^)	*D*, L g^−1^	SC metal/U
			PLS	PPLS	PPA-PGMA				
Si	SiO_2_	74.65	58 548.87	5119.76	5114.76	10.00	0.36	0.002	0.00
Al	Al_2_O_3_	13.28	4992.45	356.82	353.18	7.28	0.27	0.021	0.01
Fe	Fe_2_O_3_	3.09	7011.78	310.09	290.01	40.16	0.72	0.138	0.08
Ca	CaO	1.41	291.23	279.23	272.34	13.78	0.34	0.051	0.03
Mg	MgO	0.51	421.61	404.36	399.01	10.70	0.44	0.027	0.02
K	K_2_O	2.74	6198.21	389.61	387.34	4.54	0.12	0.012	0.01
Na	Na_2_O	1.85	4378.93	307.93	301.73	12.40	0.54	0.041	0.02
Mn	MnO	0.03	50.23	39.93	37.84	4.18	0.08	0.110	0.06
Ti	TiO_2_	0.05	47.71	46.31	45.91	0.80	0.02	0.017	0.01
U	mg L^−1^	801.43	190.03	169.43	91.87	155.12	0.652	1.688	1.00
LOI		0.47							
Total		98.16			Total	258.96	3.526		

Naturally, the 310.09 mg Fe per L iron concentration would require a selective precipitation procedure for initial removal, and the adsorption method may be applied for polishing. The sensitivity of the adsorption effectiveness to complex actual effluents including various metal ions was therefore assessed by treating the leachate with the adsorbent. As a result, iron, which may be a strong rival, surpasses the U(vi) content by around 7.78 times (5.54 mmol Fe per L and 0.712 mmol U per L, respectively). Consequently, a precipitation pre-treatment was employed, with the pH of the solution being maintained at ∼3.87. A uranium content loss of around 10.8% (or 20.62 mg U per L) is the outcome of the precipitation process. 169.4 mg U per L of residual uranium was found in the acidic pregnant fluids.

**Fig. 12:**
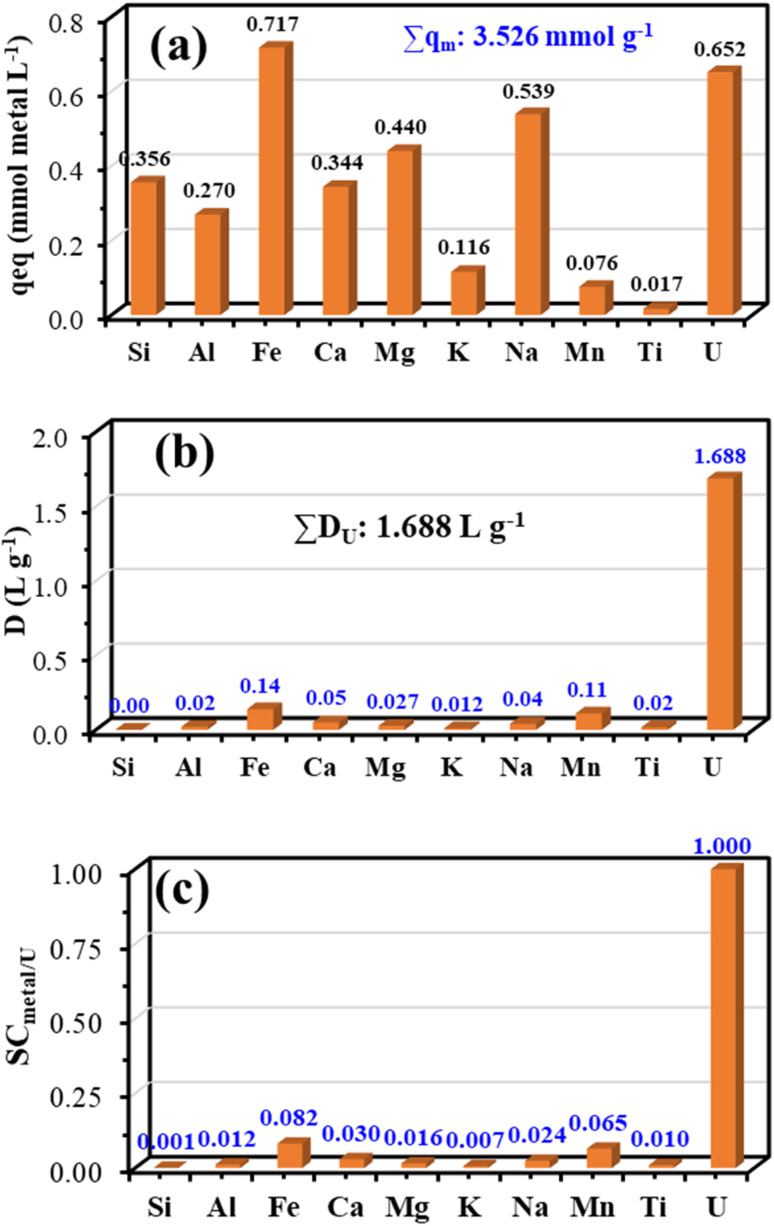
Selectivity tests: (a) adsorption capacities (in mmol metal per g), (b) distribution coefficients (*k*_d_ in mL g^−1^), and (c) selectivity coefficient (SC_metal/U_ × 10^3^) (pH_0_: 4.01; *T*: 55 ± 1 °C; SD: 0.5 g L^−1^; time: 3 h).

Three parameters—adsorption capacity at equilibrium (*q*_eq_, (a)), distribution ratio (*D*, (b)), and selectivity coefficient (SC_metal/U_, (c))—are used to illustrate the adsorption of different metal ions for PPA-PGMA (Fig. 12). SC_metal/U_ = *D*_metal_/*D*_U_ is the definition of the selectivity coefficient.^13^ U(vi) adsorption capabilities are nevertheless quite high at ∼0.65 mmol U per g and are often greater than the values seen for other metals, even with the enormous overabundance of competitor ions (Fig. 12a). Lower that of the synthetic solutions, in general. The reduction in maximum adsorption does not exceed 21.5%, regardless of the complexity of the solution and the concurrent adsorption of other metals. Common competing ions such as Ca^2+^, Mg^2+^, Si^4+^, K^+^ and Ti^4+^ generally exhibit much weaker competitive effects toward U(vi) adsorption due to their lower charge density and limited coordination ability. This behavior has been clearly demonstrated in recent literature, including the latest report by Liu *et al.*^76^ The findings in that study further support our rationale that multivalent hydrolyzing ions represent the dominant competitors in uranium-bearing leach systems.

The total metal sorption capacity is approximately 3.53 mmol g^−1^, or 258.96 mg U per g. This *q*_m_ values was substantially greater when compared to synthetic solutions: 0.828 mmol U per g. Both the large concentrations of co-metal ions and their binding to other functional groups may be responsible for this notable increase. This suggests that many reactive groups are crucial for the sorption of various metal ions.^13,16,28^

U(vi) has a distribution ratio of 1.69 L g^−1^ (Fig. 12b), which is significantly higher than the values for the other metals: ∼0.138 L g^−1^ for Fe(iii), whereas the *D* values for the other elements range from 0.002 to 0.11 L g^−1^, highlighting the higher affinity for U(vi) followed by Fe(iii) in particular (Fig. 12b). This indicates that, in contrast to its real proportion in the liquid phase, this adsorbent significantly enriches uranium in the solid phase. Unexpectedly, U(vi) exhibited superior selectivity (SC_metal/U_ was 1.0) and higher *D* values (1.69 L g^−1^) compared to other co-ions. Additionally, PPA-PGMA is more efficient and specific for U(vi) adsorption. The selectivity coefficients are shown throughout U(vi) in Fig. 12c. It is more obvious how strongly U(vi) and Fe(iii) bind to other metal ions. For very high uranium selectivity, the adsorbent therefore exhibits preferential affinity and competitiveness.

## 4. Conclusion

Polyglycidyl-methacrylate (PGMA) functionalized with polyamine and further with phosphonic acid (PPA-PGMA) was synthesized and thoroughly characterised. At 328 K and ideal pH_0_ ∼ 4.0, U's maximum adsorption capacity was 0.828 mmol g^−1^. After 180 minutes of contact, the equilibrium is reached, and it closely resembles the pseudo-second order (PSO) equation. The adsorption process is endothermic, spontaneous, and is followed by an increase in the system's unpredictability. The generalized Langmuir equation effectively fits the adsorption isotherms. With NaHCO_3_ (0.25 M), U desorption is incredibly effective and can be recycled at least six times. PPA-PGMA exhibits exceptional strength, stability, performance, durability, and utility. PPA-PGMA displayed high affinity and selectivity for U(vi), evidenced by a distribution ratio of 1.69 L g^−1^, compared to 0.138 L g^−1^ for Fe(iii) and 0.002–0.11 L g^−1^ for other ions, while the selectivity coefficients assigned U(vi) a reference SC of 1.0. These values confirm strong competitive preference toward U(vi) in acidic leach liquors. On the basis of the experimental results, a mathematical simulator is also being created and constructed using MATLAB code. The output findings and measured values were used to validate and corroborate the mathematical formula. The adsorbent has been effectively used for uranium ore acid leaching, or U recovery from precipitated pregnant liquor solution. Even with a precipitation pre-treatment, the presence of high amounts of Si and Fe lowers uranium's adsorption ability. However, the adsorption capacities of the Gattar and El Sella ore samples are around 0.65 and 0.71 mmol U per g, respectively, suggesting a decline in U adsorption efficiency of about 21.5% and 14%, respectively. Furthermore, despite its predilection for U(vi) over Fe, the adsorbent exhibits exceptional selectivity against other metal ions, as indicated by the distribution ratio and selectivity coefficient values. This micron-sized magnetic functionalized adsorbent combines fast kinetics, large sorption capacities, high regeneration potential, and relatively excellent selectivity for U(vi) against the bulk of the metal ions present in acidic leaching liquors. This material shows promise for recovering U(vi) from complicated solutions.

## Conflicts of interest

The corresponding author, representing all authors, declares that there are no conflicts of interest.

## Supplementary Material

RA-016-D5RA08591H-s001

## Data Availability

All relevant data supporting this research are included in the article. Supplementary information (SI): adsorbent synthesis and functionalization procedures, elemental and surface characterization data (SEM, XRD, FTIR, XPS, BET, and PZC analyses), adsorption kinetic and isotherm modeling, thermodynamic and diffusion parameters, regeneration studies, statistical fitting models, and additional tables and figures supporting the experimental and theoretical analyses presented in the main text. See DOI: https://doi.org/10.1039/d5ra08591h.
