# Supplementary material for: Functionalized poly(glycidylmethacrylate) for selective uranium(vi) adsorption: experimental and theoretical calculation insights
Source: RSC Adv. 2026 Feb 2;16(8):6747–67. doi: 10.1039/d5ra08591h (PMC12863290; doi:10.1039/d5ra08591h)
Supplement: RA-016-D5RA08591H-s001 [file RA-016-D5RA08591H-s001.pdf]

Supplementary Information (SI) for RSC Advances.
This journal is © The Royal Society of Chemistry 2026

Supporting Information Section

Functionalized poly(glycidylmethacrylate) for selective uranium(VI) adsorption: Experimental and theoretical calculations Insights

Ahmad A. Tolba, Ebrahium AbdelGwad, Marwa M. Rashad, Zeinab M. Shalaby, Walaa A. Kassab, Nilly A. Kawady, Said E. Mohammady, Ahmed H. Orabi*

Nuclear Materials Authority, P.O. Box 530, El-Maadi, Cairo, Egypt

*Corresponding authors: A_orabi_chem@yahoo.com

Section A – Adsorbent preparation

Section SI.

1. PGMA Preparation

Dispersion polymerization technique was applied to produce the parent poly(glycidyl methacrylate) microparticles (PGMA)[10], according to the following procedure: the dispersion medium was previously prepared by dissolving 3 g of PVP K-30 in 90 mL ethanol/water solution (90% w/w) in 250 mL four-necked flask. Then, 0.2 g of the polymerization initiator (AIBN) was dispersed in 10 g of the monomer phase (GMA) and transferred into the dispersion medium. The mixture in the polymerization reactor was subjected to nitrogen gas bubbling for 30 min to remove gas phase and dissolved oxygen. Thereafter, the polymerization reaction was carried out under reflux for 24 h at 70 °C with mechanical stirring. The obtained microspheres of PGMA were collected by centrifugation and washed thoroughly with deionized water and ethanol, and finally dried under vacuum at ambient temperature.

2. PGMA Functionalization

The polyaminophosphonic acid-functionalized polyglycidyl methacrylate was prepared through two sequential stages[11]. In the first one, the previously prepared PGMA microspheres (10 g) were suspended in ethanol (20 mL) followed by addition of diethylenetriamine (DETA, 12 mL), then the reaction mixture was stirred for 18 h under reflux. The aminated PGMA was collected and recovered through filtration and repeatedly washed. In the second stage, phosphorous acid (5 g) was dissolved in 100 mL of HCl/water solution (1:1, v/v) followed by addition of the aminated PGMA (1 g), the mixture was then heated and refluxed in a 200 mL three-necked flask supplied with dropping funnel, thermometer and condenser. The formaldehyde solution (20 mL) was added dropwisely during 1 h and the mixture was kept under reflux for the next 24 hours [8]. The final product of polyaminophosphonated-PGMA was collected via filtration and extensively washed with ethanol and water. Finally, the sorbent was dried for 24 h at 75 °C.

Table S1. Elemental analysis and PZC values for PGMA, NH_2-PGMA, and PPA-PGMA.

Material		C (%)	C (mmol g^{-1})	H (%)	N (%)	N (mmol g^{-1})	P (%)	P (mmol g^{-1})	O (%) *	O (mmol g^{-1})	PZC
PGMA	Aver.	57.8	48.13	7.29	0.28	0.2	0	-	34.63	21.64	5.81
	S.D.	0.03		0.07	0.05						
NH_2-PGMA	Aver.	42.81	35.65	7.71	12.56	8.97	0	-	36.92	23.08	8.49
	S.D.	0.08		0.09	0.03						
PPA-PGMA	Aver.	34.69	28.88	7.06	9.36	6.68	5.16	1.67	43.73	27.33	2.65
	S.D.	0.09		0.11	0.04		0.15				

*: obtained by difference to 100 % (w/w fraction); n.d.: not determined.

Table S2. Adsorption modeling of uptake kinetics and sorption isotherms [2].

Operation	Model	Equation	Parameters	
Kinetics	PFORE	$q(t) = q_{eq,1}(1 - e^{k_1 t})$	$q_{e,1}$ (mmol/g)	k_1 (min^{-1})
	PSORE, Non-linear form	$q(t) = \frac{q_{eq,2}^{2} \times k_2 \times t}{1 + q_{eq,2} \times k_2 \times t}$	$q_{e,2}$ (mmol/g)	k_2 (L/mmol.min)
	PSORE, Llinear form	$\text{Log } (q_e - q_t) = \log q_e - (k_1/2{:}303) * t$		
	sRIDE<br>(Weber & Morris)	$q_{(t)} = k_{int.,i} \cdot t^{0.5} + C$<br>Several linear sections corresponding to different regimes of resistance (i) to intraparticle diffusion may co-exist ($K_{int,i}$) (linear regression calculation)	$K_{int.i}$ (mmol/g.$min^{-0.5}$)	
Isotherms	Langmuir, Non-linear form	$q_{eq} = \frac{q_{m,L} \times b_L \times C_{eq}}{1 + b_L \times C_{eq}}$	$q_{m,L}$ (mmol/g)	b_L (L/mmol)
	Freundlich, Non-linear form	$q = k_F C_{eq}^{1/n_F}$	k_F	n_F (dimensionless)
	Langmuir, Linear form	$\frac{C_{eq}}{q_{eq}} = \frac{C_{eq}}{q_{max}} + \frac{1}{bq_{max}}$	q_{max} (mmol/g)	b_L (L/mmol)
	Freundlich, Linear form	$\text{Log } q_e = \log k_f + 1/n_f \log C_e$	k_F	n_F (dimensionless)

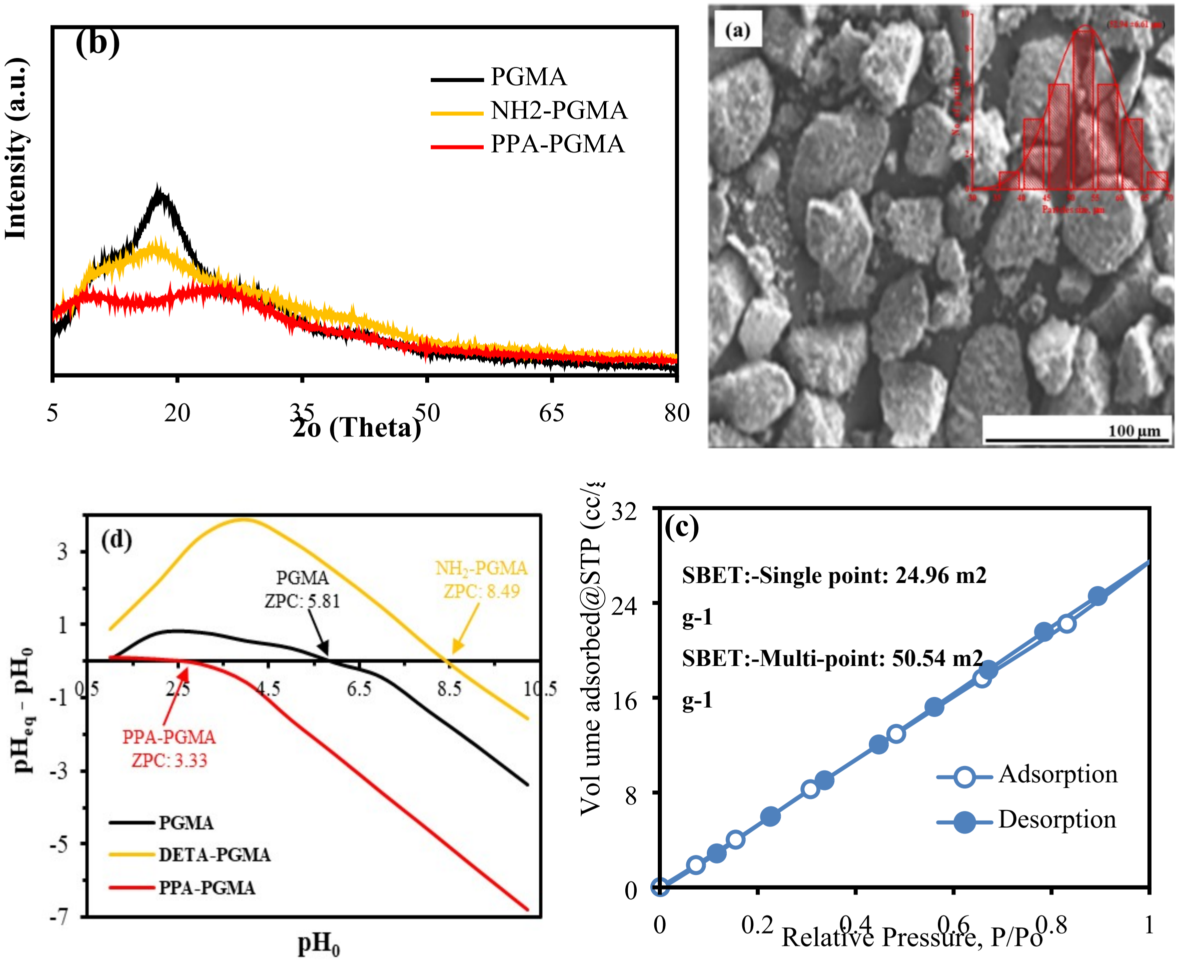

Fig. S1. SEM image and particle size analysis for PGMA (a), XRD patterns for PGMA, NH_2-PGMA and PPA-PGMA materials (b), Textural analysis of P-PGMA–N_2 adsorption and desorption isotherms, and ZPC analysis (d).

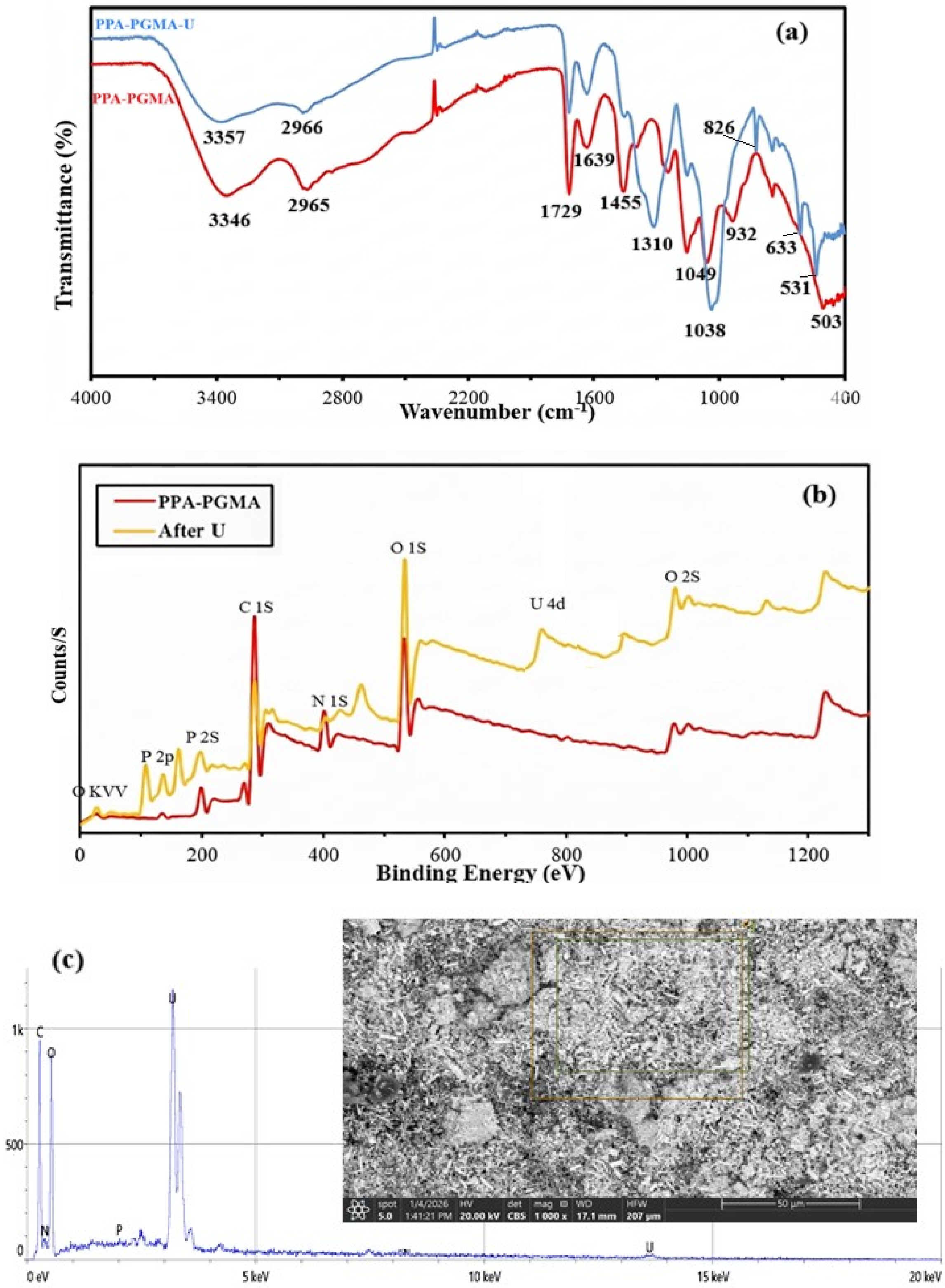

Fig. S2. FTIR spectrum for PPA-PGMA after uranium adsorption (a), XPS survey for PPA-PGMA after uranium adsorption (b), and SEM-EDS analysis for PPA-PGMA after uranium adsorption (c).

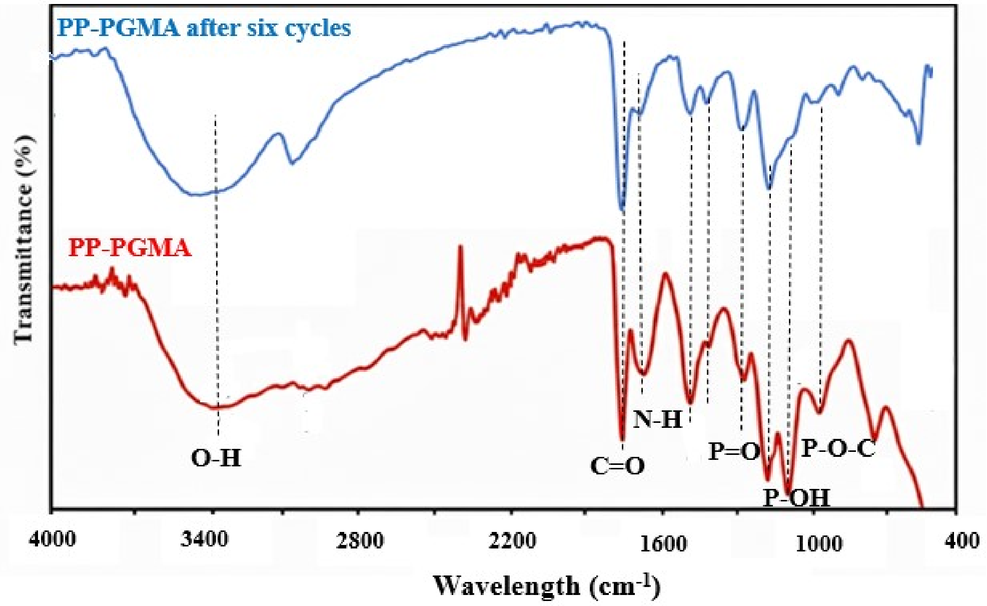

Fig. S3. FTIR spectrum for PPA-PGMA after six cycles of uranium adsorption/desorption.

Table S3. Different probabilities of variables of Pseudo 2nd order model.

t	T	qtUexp	qtUcalc	error%	qtUcor	error%
**5**	299.15	0.2639	0.2641	-0.080	0.265	-0.4398
**15**	301.79	0.3744	0.3753	-0.240	0.369	1.4343
**30**	304.42	0.4747	0.4855	-2.280	0.478	-0.6341
**45**	307.06	0.5945	0.5644	5.060	0.591	0.5514
**60**	309.70	0.6352	0.6212	2.210	0.635	-0.0144
**90**	312.33	0.6748	0.6932	-2.730	0.671	0.5035
**120**	314.97	0.7286	0.7315	-0.390	0.731	-0.3786
**180**	317.60	0.7738	0.7635	1.340	0.769	0.6124
**240**	320.24	0.7746	0.7727	0.200	0.775	-0.0275
**300**	322.88	0.7759	0.7755	0.052	0.776	-0.0198
**360**	325.51	0.7770	0.7763	0.080	0.774	0.0717
**480**	328.15	0.7767	0.7767	0.003	0.777	0.0209

Table S4. Different probabilities of variables of Two reactions with Arrhenius constants and the activation energies, Shrinking core Model, and Thermodynamics parameters (Parameters, constants and goodness of fit).

**Two reactions with Arrhenius constants and the activation energies**		**Shrinking Core Model**		**Thermodynamics parameters**	
parameters	Values	Parameters	Values	parameters	Values
Ar_1	0.0086	Ar_1	0.0086	m	5.46
Ar_2	0.3254	Ar_2	0.3254	n	5.163
ΔE_1**	2.3149	ΔE_1**	2.3150	e	4.534
ΔE_2**	1.2928	ΔE_2**	1.2930	F	5.246
a	0.556	kC	9592	g	1.05
qm***	0.7767	kD	0.0055	qm***	0.7739
SSE	0.0001	Ds	5.92E-10	ΔH*	6.0948
R^2	0.9998	kf'	12710	ΔS, KJ/mol.K	80.6799
AdjR^2	0.9973	qm***	0.7767	ΔG**, 299	-18.0406
RMSE	0.0091	SSE	517.90	ΔG**, 308	-18.7667
		R^2	0.9819	ΔG**, 318	-19.5735
		AdjR^2	0.9759	ΔG**, 328	-20.3803
		RMSE	9.2910	SSE	0.0015
				R^2	0.9939
				AdjR^2	0.9878
				RMSE	0.017

* 'kC': solid diffusion controlled, kD: solid diffusion controlled, Ds, solid diffusivity, cm/s.
Units: **: KJ/mol, ***: mmol/L.

Table S5. Different probabilities of variables of Floatotherm model.

**pH_0**	**C_0, mmol/L**	**q_{exp} mmol/g**	**q_{calc1} mmol/g**	**Error%**	**T, K**	**$q_{calc\ 2}$ mmol/g**	**Error%**	**q_{cor}**	**Error%**
2.01	0.313	0.246	0.246	-0.02	299	0.246	-0.019	0.247	-0.487
3.04	0.318	0.400	0.390	2.59	302	0.390	2.588	0.395	1.138
4.01	0.311	0.405	0.4021	0.83	304	0.402	0.845	0.408	-0.774
5.01	0.318	0.407	0.406	0.19	307	0.406	0.233	0.413	-1.402
6.00	0.313	0.408	0.402	1.47	310	0.402	1.526	0.408	-0.082
4.01	0.193	0.281	0.288	-2.35	313	0.288	-2.509	0.279	0.853
4.00	0.362	0.438	0.431	1.56	316	0.431	1.629	0.436	0.386
4.01	0.548	0.505	0.514	-1.69	319	0.513	-1.590	0.499	1.295
4.01	0.730	0.545	0.577	-5.99	322	0.577	-5.861	0.551	-1.104
4.01	1.073	0.672	0.674	-0.32	325	0.673	-0.186	0.670	0.345
4.01	1.429	0.766	0.757	1.26	328	0.756	1.403	0.767	-0.070

Table S6: The chemical analysis Composition major and traces elements of the El-Sella ore.

Major oxides,	SiO_2	Al_2O_3	Fe_2O_3	CaO	Na_2O	MgO	K_2O	P2O5	TiO_2	L.O.I	Total
Wt., %	69.93	13.13	5.22	1.81	0.34	0.65	1.82	0.69	1.09	5.41	99.78
Trace	U	Th	REEs	Zr	Y	Rb	Nb	Sr	Pb		
mg L^{-1}	1173.4	22	530.4	294.2	53.8	198.2	123.8	1048	276.8		

Wt: weight percentage; LOI: Loss on ignition.

Table S7. The chemical analysis Composition major and traces elements of the granite sample.

Major oxides,	SiO_2	Al_2O_3	Fe_2O_3	CaO	Na_2O	MgO	K_2O	P2O5	TiO_2	L.O.I	Total
Wt., %	74.65	13.28	3.06	1.4	1.85	0.5	2.74	0.04	0.05	0.47	98.04
Trace	U	Th	REEs	Zr	Y	Cr	Nb	Zn	Pb	Ba	Ga
mg L^{-1}	801.43	190	219	343	149	179	92	317	397	1425	139

Reference

[8] A.A. Galhoum, W.H. Eisa, I. El-Tantawy El-Sayed, A.A. Tolba, Z.M. Shalaby, S.I. Mohamady, S.S. Muhammad, S.S. Hussien, T. Akashi, and E. Guibal, A new route for manufacturing poly(aminophosphonic)-functionalized poly(glycidyl methacrylate)-magnetic nanocomposite - Application to uranium sorption from ore leachate. Environmental Pollution, 2020, **264**, 114797.

[10] A.A. Galhoum, T. Akashi, M. Linnolahti, J.T. Hirvi, A.G. Al-Sehemi, A. Kalam, and E. Guibal, Functionalization of poly(glycidylmethacrylate) with iminodiacetate and imino phosphonate groups for enhanced sorption of neodymium - sorption performance and molecular modeling. Reactive and Functional Polymers, 2022, **180**, 105389.

[11] A.A. Galhoum, E.A. Elshehy, D.A. Tolan, A.M. El-Nahas, T. Taketsugu, K. Nishikiori, T. Akashi, A.S. Morshedy, and E. Guibal. Synthesis of polyaminophosphonic acid-functionalized poly(glycidyl methacrylate) for the efficient sorption of La(III) and Y(III), Chemical Engineering Journal, 2019, **375,** 121932.
